# Oxygen Isotope Analyses of Phosphate and Organophosphorus Compounds by Electrospray Ionization Orbitrap Mass Spectrometry

**DOI:** 10.1021/acs.analchem.5c04367

**Published:** 2025-10-10

**Authors:** Nora M. Bernet, Cleo Soldini, Timo M. O. Felder, Kristýna Lapčíková, Cajetan Neubauer, Wendy L. Queen, Ralf Kaegi, Federica Tamburini, Thomas B. Hofstetter

**Affiliations:** † 28499 Eawag: Swiss Federal Institute of Aquatic Science and Technology, 8600 Dübendorf, Switzerland; ‡ Institute of Biogeochemistry and Pollutant Dynamics (IBP), ETH Zürich, 8092 Zürich, Switzerland; § Institute of Chemical Science and Engineering (ISIC), 27218 École Polytechnique Fédérale de Lausanne (EPFL), 1951 Sion, Switzerland; ∥ University of Chemistry and Technology, Prague (UCT Prague), 166 28 Prague, Czech Republic; ⊥ 1877 University of Colorado Boulder, Boulder, Colorado 830303, United States; # Institute of Agricultural Sciences, ETH Zürich, 8315 Lindau, Switzerland

## Abstract

Orbitrap mass spectrometry with electrospray ionization (ESI-Orbitrap MS) enables ^18^O/^16^O ratio measurements in phosphate and organophosphorus compounds, which offers promising avenues for the study of metabolic, biogeochemical, and environmental processes. While the instrumental feasibility of such ^18^O/^16^O ratio measurements has been shown, the applicability of such analyses in aqueous matrices on multipurpose mass spectrometers remains unaddressed. Here, we (i) evaluated the interplay between instrument parameters and the long-term accuracy and precision of ESI-Orbitrap MS for the determination of δ^18^O­(PO_4_) from H_2_PO_4_^–^ vs PO_3_^–^ fragments of phosphate, (ii) identified properties of methanolic sample solutions that are critical for accurate and precise measurements, and (iii) propose a sample purification procedure for the elimination of matrix-based interferences. In 10 measurement campaigns over two years, we observed a long-term reproducibility of δ^18^O­(PO_4_) within ±2.8‰. Results of in-source fragmentation experiments of H_2_PO_4_^–^ to PO_3_^–^ show excellent agreement of δ^18^O and offer promising opportunities to probe for ^18^O/^16^O ratios in organophosphorus compounds. By investigating the effect of the aqueous matrix and interfering anions on ^18^O/^16^O ratio measurements, we found that a water content exceeding 50 vol % and the presence of oxyanions such as nitrate and sulfate limit measurement accuracy due to interferences of matrix constituents in the ESI source. To overcome these challenges, we evaluated selective phosphate extraction with a zirconium-based metal–organic framework (MOF) as sorbent. The resulting purification procedure allowed for successful extraction and recovery of phosphate from nitrate- and sulfate-containing aqueous solutions, resulting in methanolic phosphate samples that enabled accurate analyses of δ^18^O­(PO_4_) by ESI-Orbitrap MS.

## Graphical abstract

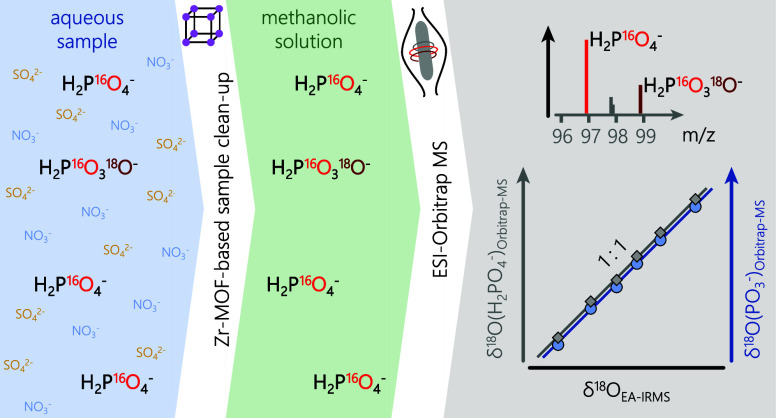

## Introduction

Orbitrap mass spectrometry (Orbitrap MS) offers new avenues to determine natural abundance stable isotope ratios of numerous elements in diverse compounds of bio­(geo)­chemical and environmental interest.^1–9^ The ability to introduce intact molecules into the Orbitrap (Fourier transform) mass spectrometer substantially extends the current capabilities of established isotope ratio analysis by isotope ratio mass spectrometry (IRMS), nuclear magnetic resonance, and laser absorption spectroscopy. Quantification of abundances of whole-molecule isotopologues and/or mass fragments thereof by Orbitrap MS enables quantification of isotope ratios of several elements simultaneously and in a position-specific manner. Moreover, a direct introduction of solutions containing polar or ionic analytes, including oxyanions, amino acids, and small organic acids, could potentially simplify the sometimes tedious sample preparation workflows for bulk and compound-specific IRMS. Indeed, the perspective of higher sample throughput and a wider range of analytes could promote applications of stable isotope analysis. The required Orbitrap MS instrumentation is often available in mass spectrometry facilities for chemical, biological, and environmental analyses and can be dedicated to isotopic analysis without instrumental modifications.

The analysis of oxygen isotope ratios in phosphate and organophosphorus compounds for studying metabolic and biogeochemical processes in the environment and deciphering pollutant transformation dynamics would indeed benefit from advances in Orbitrap MS-based analytical procedures. In fact, variations in ^18^O/^16^O ratios from phosphoryl transfer reactions, that is, the nucleophilic displacement of PO_3_^2–^ groups between phosphate esters and water, have long been exploited to study enzyme mechanisms.^10–14^ Observations of ^18^O/^16^O ratios in phosphate from various environments^15–18^ have since been proposed as markers for metabolic activity or changes thereof. Furthermore, controversies about sources of water pollution with the herbicide glyphosate and its biodegradation products from agricultural use vs aminopolyphosphonates used in laundry detergents^19^ could potentially be resolved from source apportionment with ^18^O/^16^O ratios used as fingerprints for phosphonate moieties. Despite the known and hypothesized utility of information from ^18^O/^16^O ratio evaluations in phosphate and organophosphorus compounds, applications of the O isotope ratio analysis are rare. So far, standard methods for the determination of ^18^O/^16^O ratios in phosphate and organophosphorus compounds rely on bulk isotope ratio analysis by elemental analysis (EA) and IRMS.^20^ However, these techniques cannot distinguish between different O-bearing moieties of the analyte. While this approach enables highly precise measurements of the ^18^O/^16^O ratios, it requires isolation of the analyte PO_4_^3–^ as Ag_3_PO_4_ salts for the O isotope analysis of phosphate.^21,22^ These boundary conditions imposed on the isolation and purification of the analyte severely limit widespread applications of ^18^O/^16^O ratio analyses of phosphate and organophosphorus compounds.

If used in combination with electrospray ionization (ESI-Orbitrap MS), this technique offers new avenues to overcome some of the limitations of the ^18^O/^16^O ratio analysis of phosphate.^3,23^ Recent studies illustrate the feasibility of ^18^O/^16^O ratio measurements of PO_4_^3–^ with precision δ^18^O < 1‰ using nanomolar solutions of phosphate. The perspective to carry out accurate measurement in PO_3_^–^ fragment isotopologues instead of those of the original H_2_PO_4_^–^ ions^23^ also hints at the opportunity to obtain position-specific δ^18^O values, for example, in phosphate and phosphonate moieties of organophosphorus compounds. To enable widespread applications of ^18^O/^16^O ratio analysis of these compounds in biological and environmental matrices and thus capitalize on current instrumental achievements, critical steps in sample preparation need to be addressed. Current data are based solely on the analysis of phosphate ions dissolved in methanol, while phosphate samples typically arise as aqueous solutions. Moreover, solutions of phosphate and organophosphorus compounds also contain cations, and very likely, other (oxy)­anions whose interferences on accuracy and precision of ^18^O/^16^O ratio determination of phosphate require thorough assessment (see, e.g., the potential of isobaric interferences from HSO_4_^–^ in Wang et al.^23^). It is, in fact, well-known that typical cations (e.g., Na^+^, K^+^, NH_4_^+^, and Ca^2+^) and anions (e.g., NO_3_^–^, Cl^–^, and SO_4_^2–^) in aqueous sample matrices can compromise the ionization of the target analyte and lead to adduct formation.^24,25^ The extent to which the ionic composition of samples affects the accuracy and precision of ^18^O/^16^O ratio measurements beyond expected consequences for sensitivity is poorly known. Thus, sample preparation and cleanup strategies to mitigate such effects of aqueous matrices remain to be developed.

The goal of this study was to develop a procedure that allows for ^18^O/^16^O ratio measurements of phosphate from aqueous matrices using standard ESI-Orbitrap MS instrumentation. Specifically, we (i) evaluated critical instrument operating parameters for repeated ^18^O/^16^O ratio determination over a period of two years. In addition, we tested the known opportunities of PO_3_^–^-fragment-based O isotope ratio analysis of phosphate^23^ and organophosphorus compounds, which we explored using substances that represent typical phosphonate pollutants in wastewater streams. (ii) We assessed the properties of methanolic sample solutions that are critical for accurate and precise measurements. (iii) Finally, we developed a sample cleanup procedure to manage ionic compositions of sample solutions prior to mass spectrometric analyses. To that end, we explored the use of metal–organic frameworks (MOFs) as sorbent materials for the selective extraction of phosphate from aqueous solutions. MOFs have indeed been shown to extract phosphate rapidly and selectively over common anions in natural waters.^26–31^ However, the use of MOFs as sorbent materials and the quantitative recovery of phosphate for isotopic analyses have not been studied so far. To that end, we investigated the extraction of phosphate as an analyte by MOFs, its recovery therefrom, and the consequences of MOF-based procedures for further sample treatment prior to ^18^O/^16^O ratio measurements.

## Experimental Section

### Chemicals and Isotopic Standards

A full list of chemicals and materials used can be found in Section S1.1 of the Supporting Information (SI).

Laboratory working standards of phosphate with known δ^18^O­(PO_4_) values were prepared following the procedure in Lécuyer et al.^22^ which has been established for the use of the Ag_3_PO_4_ reference material.^21,32^ Potassium dihydrogen phosphate (KH_2_PO_4_) was dissolved in nanopure water, spiked with different amounts of ^18^O-labeled water (H_2_^18^O), and heated to 120 °C for 21 days in sealed Pyrex vials. The samples were quenched in a cold water bath, and a cleaning procedure^33^ using AW 50W cation exchange resin was applied. The obtained phosphate isotopic working standards were split into two fractions. The first fraction was prepared for ^18^O/^16^O ratio measurements by EA-IRMS according to the Ag_3_PO_4_ precipitation protocol by Tamburini et al.^33^ and analyzed by EA-IRMS (δ^18^O­(PO_4_) listed in Table S1). The second fraction was diluted to a final concentration of 50 μM with methanol (MeOH) for analysis by ESI-Orbitrap MS. Phosphate working solutions were prepared by dissolving anhydrous sodium dihydrogen phosphate (NaH_2_PO_4_) in nanopure water, followed by dilution using methanol to a target concentration between 5 and 50 μM.

### Zr-Based Metal–Organic Framework (Zr-MOF)

Zr-BDC (Zr-1,4-benzenedicarboxylate), also known as UiO-66, was synthesized according to a previously reported method by Katz et al.^34^ In short, ZrCl_4_ (2.41 g, 1 equiv) was dispersed in 180 mL of *N,N*-dimethylformamide (DMF) in a 1 L cap-screw vessel, and 18 mL of HCl (37 wt %) was added. Terephthalic acid (2.22 g, 1.29 equiv) was dissolved in 360 mL of DMF, sonicated, and added to the mixture. The reaction mixture was sonicated for 15 min, followed by heating at 80 °C for 24 h. After the reaction was allowed to cool to room temperature, the crude product was placed in 50 mL centrifugation vessels, washed 3 times with 40 mL of DMF, and solvent exchanged with ethanol (3 × 40 mL, overnight). The final product was dried at 80 °C under vacuum for 24 h, yielding Zr-BDC as a white crystalline solid. The material was characterized by powder X-ray diffraction (PXRD), N_2_ adsorption, and thermogravimetric analysis (TGA), as described in Section S1.2. Stock suspensions of Zr-BDC were prepared in a 0.1 mM sodium chloride (NaCl) solution.

### Chemical and Isotopic Analysis

Concentrations of aqueous inorganic phosphate and sodium were quantified by UV/vis spectrophotometry and ion chromatography, respectively, following the standard methods described in Section S1.3.

^18^O/^16^O ratio measurements by ESI-Orbitrap MS were carried out on a Q-Exactive Plus Hybrid Quadrupole-Orbitrap mass spectrometer with an Ion Max atmospheric pressure ionization (API) source connected to a heated ESI probe (HESI-II; all Thermo Fisher Scientific). The instrument was equipped with a low-flow needle for flow rates of 1–10 μL/min. For sample introduction by direct infusion, a digitally controlled syringe pump (Fusion 100T, Chemyx) equipped with a 500 μL Hamilton syringe was used to deliver phosphate solutions at a flow rate of 4 μL/min. For sample loading in flow-injection mode, an UltiMate 3000 HPLC system (Thermo Fisher Scientific) equipped with an autosampler (PAL HTC-xt System) was coupled to the mass spectrometer. Infusion time periods of 50 μM methanolic phosphate solutions were 15 min. A full description of the instrument setup can be found in Section S1.4

The following ESI settings were defined based on earlier work by Neubauer et al.^3^ as a sheath gas flow rate of 2, an auxiliary gas flow rate of 1, a sweep gas flow rate of 0, spray voltage ∼2.8 kV in negative ionization mode, a capillary temperature of 290 °C, S-lens RF level 60, and an auxiliary gas heater temperature of 40 °C. Typical settings of mass spectrometric data acquisition, unless stated otherwise, were “full MS” scan type with a scan range of *m*/*z* 95–105. The number of microscans was 1, the mass resolution was 70,000, and the automatic gain control (AGC) target was 2 × 10^5^, with a maximum injection time of 1000 ms. The identical ESI and MS settings were applied for the quantification of δ^18^O­(PO_3_^–^) from phosphate mass fragments by in-source collision-induced dissociation (CID) and high-energy collision dissociation (HCD). For CID, a scan range of *m*/*z* 76–86 and CID energies ranging from 10 to 90 eV were used. For HCD, the same scan range *m*/*z* 76–86 was applied, while the precursor isolation window was set at *m*/*z* 95–105. Collision energies ranging from 10 to 80 eV and normalized collision energies between 10 and 200 were tested.

To compare Orbitrap MS results to the traditional methods, oxygen isotope analysis of our laboratory working standards in Ag_3_PO_4_ was also performed by a thermal conversion EA instrument coupled to an IRMS (EA-IRMS; Pyro Cube connected in continuous flow mode to Isoprime 100, Elementar Analysensysteme GmbH, Langenselbold, Germany). IRMS results were calibrated against IAEA benzoic acid standards 601, 602, and an internal Ag_3_PO_4_ standard (δ^18^O = +14.2‰), with a standard deviation lower than 0.4‰. δ values are expressed in the ‰-notation against Vienna Standard Mean Ocean Water (VSMOW).

### Data Evaluation

We used the IsoX application (Isotopocule data eXtraction from Orbitrap RAW files; version 2022; distributed via Thermo Fisher Scientific) for extracting relevant ion intensities from RAW files that are generated by the Orbitrap MS control software.^3,7,35^ Then, the obtained .isox files were evaluated in the IsoXL web-based graphical user interface (isoorbi.shinyapps.io/IsoXL, Version 0.53) to obtain ^18^O/^16^O ratios (see Section S1.5 for parameters used).^35^ Further data evaluation and calculations of ^18^O/^16^O ratios and the corresponding O isotope signatures, δ^18^O values, were performed in Microsoft Excel. The raw δ^18^O values from Orbitrap MS measurements in the ‰-notation were obtained using eq 1.

δ18Oraw=(Rsmp18Rref18−1)·1000 1

where ^18^ *R*_smp_ and ^18^ *R*_ref_ are the ^18^O/^16^O ratios from the quantification of ^1^H_2_P^18^O^16^O_3_^–^/^1^H_2_P^16^O_4_^–^ ratios of samples and in-house KH_2_PO_4_ isotope standard D (Table S1), respectively, by Orbitrap MS.

It should be noted that we use the following terminology to pinpoint the meaning of δ^18^O in this study. δ^18^O­(PO_4_) stands for general O isotope signatures of phosphate, regardless of the instrumental approach applied for ^18^O/^16^O ratio measurements (i.e., both Orbitrap MS and EA-IRMS). δ^18^O­(H_2_PO_4_^–^) specifies δ^18^O values obtained from measurements of the O isotopologue ratios for H_2_PO_4_^–^ by Orbitrap MS. Finally, δ^18^O­(PO_3_^–^) represents O isotope signatures obtained by Orbitrap MS from the fragmentation of H_2_PO_4_^–^ or organophosphorus compounds to PO_3_^–^.

δ^18^O­(PO_4_) values from ^18^O/^16^O ratio measurements by Orbitrap MS relative to VSMOW were derived using eq 2.

$$\delta^{18}O\left({\mathrm{PO}}_{4}\right)=\delta^{18}O_{\mathrm{raw}}+\delta^{18}O_{\mathrm{EA‐IRMS}}+\frac{\delta^{18}O_{\mathrm{raw}}\cdot \delta^{18}O_{\mathrm{EA‐IRMS}}}{1000}$$ 2

where δ^18^O_EA‑IRMS_ represents the δ^18^O­(PO_4_) value of phosphate isotope standard D quantified by EA-IRMS, and δ^18^O_raw_ stands for raw δ^18^O values derived by Orbitrap MS measurements using eq 1. We evaluated the utility of 1- vs 2-point calibrations for the derivation of δ^18^O­(PO_4_) with isotope standard E (δ^18^O_EA‑IRMS_ = −26.6‰) and G (+41.0‰) following procedures of Gröning^36^ and Hilkert et al.,^7^ as described in Section S1.6.

Measurement sequences in direct-infusion mode typically consisted of triplicate injections of isotopic standard, eight triplicates of sample solution, followed by another triplicate of isotopic standard. Sequences in the flow-injection mode were run in similar manner but included only three instead of eight triplicate sample injections. No corrections for instrumental drift (−0.23‰/h) and memory effects (0.16 ± 1.85‰) were required as both effects were negligible on the time scales of our measurement campaigns (Figure S5).

## Results and Discussion

### Long-Term Accuracy and Precision

Following the protocol proposed by Kantnerová et al.,^35^ we determined the optimal instrument settings to quantify the ratio of ^18^O and ^16^O isotopologues of phosphate accurately and precisely with a Q-Exactive Plus Orbitrap mass spectrometer. As will be discussed in detail below, we evaluated four previously identified, critical operating parameters,^3,5,7,35^ namely, (1) sample introduction and ionization parameters, (2) quadrupole mass filter range, (3) AGC target, and (4) mass resolution. Figure 1a shows the δ^18^O­(PO_4_) values of six laboratory working standards determined by direct infusion Orbitrap MS—with a flow rate of 4 μL/min, a sample injection time of 15 min, a scan range of *m*/*z* 95–105, an AGC target of 2 · 10^5^, and a mass resolution of 70,000—vs the result of conventional O isotope analysis by EA-IRMS. Over a period of 22 months and 10 measurement campaigns, we observed excellent agreement between ^18^O/^16^O ratio measurements by Orbitrap MS and EA-IRMS, covering a range of more than 90‰. The correlation of the δ^18^O values is linear (slope: 1.006 ± 0.030) with an insignificant offset (y-intercept: −1.012 ± 1.152) of δ^18^O­(H_2_PO_4_^–^) from δ^18^O­(PO_4_) obtained from the reference measurements by EA-IRMS (δ^18^O­(PO_4_)_EA‑IRMS_). This type of correlation of ^18^O/^16^O ratio measurements by ESI-Orbitrap MS and EA-IRMS was also used to evaluate the utility of 1- vs 2-point calibrations for the derivation of δ^18^O­(PO_4_) values. As illustrated in Section S1.6 for four data sets obtained between May 2023 and August 2024, data from 1-point calibrations consistently yielded correlation slopes of δ^18^O­(H_2_PO_4_^–^) vs δ^18^O_EA‑IRMS_ close to unity within 0.009 ± 0.022. Two-point calibrations showed either larger deviations or 5-fold lower precision. To that end, all δ^18^O­(H_2_PO_4_^–^) reported in the following were obtained through the 1-point referencing procedure from eq 2.

**Figure 1:**
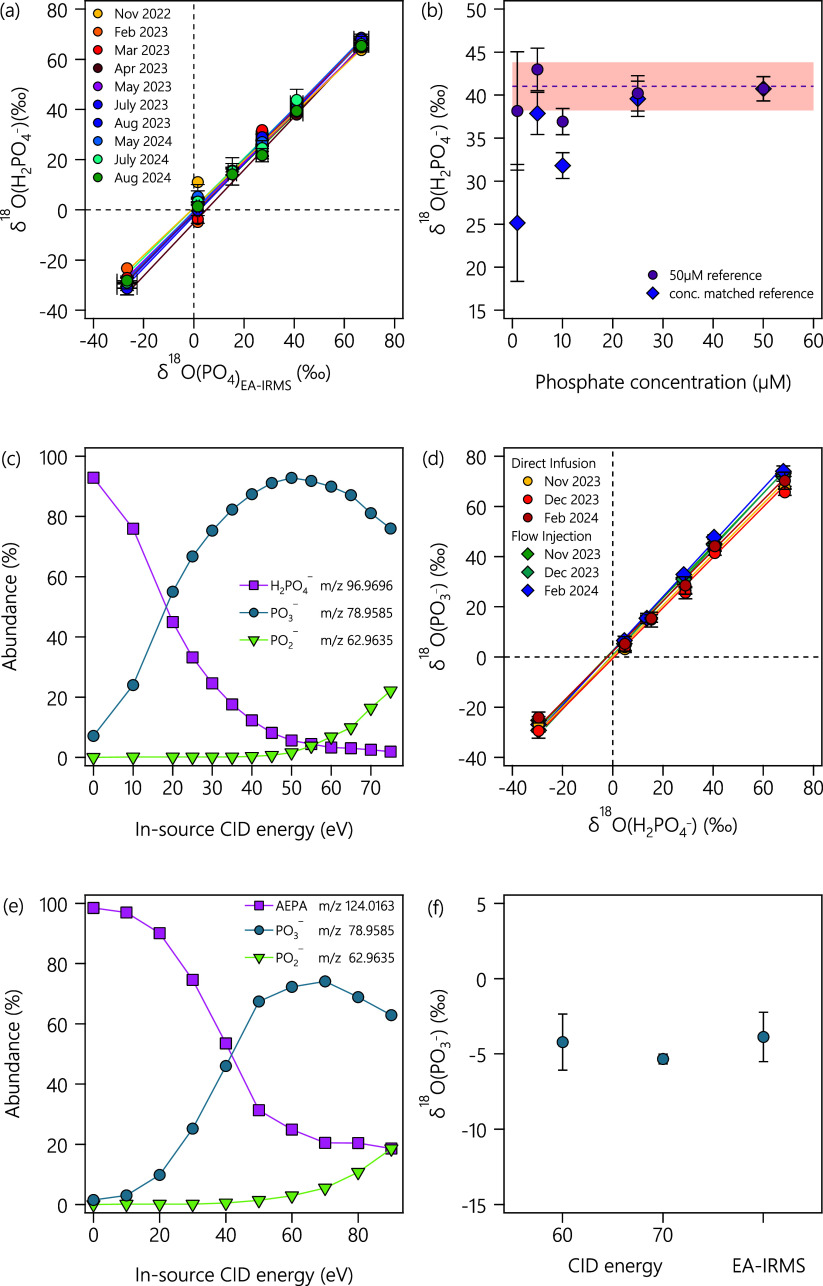
(a) δ^18^O­(PO_4_) values obtained from ^18^O/^16^O ratio measurements by Orbitrap MS (δ^18^O­(H_2_PO_4_^–^)) and EA-IRMS (δ^18^O­(PO_4_)_EA‑IRMS_) of six in-house KH_2_PO_4_ standards. The linear fit corresponds to *y* = (1.006 ± 0.030)*x* – (1.012 ± 1.152) with uncertainties corresponding to 95% confidence intervals (see Table S3 for the data). (b) Phosphate concentrations vs δ^18^O­(PO_4_) and an experimental uncertainty band of ±2.80‰ from 22-month/10 campaigns of ^18^O/^16^O ratio measurements by Orbitrap MS. Circles and diamonds refer to the concentration of the reference standard (50 μM vs concentration of a sample). The concentration of phosphate in the reference standard was set to 50 μM in this study. (c) Relative abundances of H_2_PO_4_^–^, PO_3_^–^, and PO_2_^–^ species at different in-source collision-induced dissociation (CID) energies. (d) Correlation of δ^18^O values of six laboratory working standards determined in the absence and presence of in-source fragmentation (45 eV) as δ^18^O­(H_2_PO_4_^–^) and δ^18^O­(PO_3_^–^), respectively. Linear correlation fits were *y* = (0.976 ± 0.003)*x* + (0.762 ± 1.429) for measurements performed in direct-infusion mode and *y* = (1.038 ± 0.019)*x* + (2.499 ± 1.018) for measurements performed in flow-injection mode. (e) Relative abundances of ion intensities of (2-aminoethyl)­phosphonic acid (AEPA), PO_3_^–^, and PO_2_^–^ at different in-source CID energies. (f) δ^18^O­(PO_4_) values determined for PO_3_^2–^ from AEPA at the two CID energies by Orbitrap MS and measured by EA-IRMS.

We observed an identical linear relationship over six months (five measurement campaigns) when ^18^O/^16^O ratios were measured by flow-injection analysis (Figure S4). The same quality of linear correlation was found previously between ^18^O/^16^O ratio measurements by Orbitrap MS and IRMS over approximately 80‰ of δ^18^O for nitrate and sulfate.^7^ Conversely, previous Orbitrap MS-based ^18^O/^16^O ratio determination of phosphate showed larger deviations from linearity (6% over a δ^18^O­(PO_4_) range of 20‰) and a substantial offset (20‰).^23^

### Orbitrap MS Operating Parameters

Using the optimized ESI source settings summarized above, we evaluated the effect of sample infusion time on the relative standard error of ^18^O/^16^O ratio measurements. Figure S8a shows that the relative standard error or acquisition error of ^18^O/^16^O ratio measurements decreases from 2.4‰ at 5 min to 1.0‰ after 30 min and 0.7‰ after 60 min for measurements at a mass resolution *R* of 70,000. Decreasing the relative standard error over this time period corresponds to increasing sample volumes to >250 μL per injection. The results illustrate how extended data acquisition periods, that is, measurement times on the Orbitrap MS instrument, enable precise ^18^O/^16^O measurements following the dependence on ion-counting statistics (Figure S6), as illustrated previously.^7,23^ However, an implementation of extended data acquisition periods will be limited by available sample volumes and/or requirements for sample throughput.

We tested four different quadrupole *m*/*z* ranges in which the two relevant phosphate isotopologues, ^1^H_2_^31^P^16^O_4_^–^ (*m*/*z* = 96.970) and ^1^H_2_^31^P^18^O^16^O_3_^–^ (*m*/*z* = 98.974), were quantified, while other potentially interfering ions generated in the ESI source were excluded (Table S4). Relative standard errors were only moderately sensitive to *m*/*z* ranges between 4 and 20 Da. These errors were the smallest (1.39 ± 0.02) for the *m*/*z* range of 95–105 and increased by 0.45‰ as the *m*/*z* range decreased to 95.5–99.5. This observation is consistent with previous findings and the interpretation that ion selection with the quadrupole mass filter can bias ion intensity ratios due to space-charge effects on ion transmission.^1,37^

We investigated the consequence of varying AGC targets between 2 × 10^4^ and 5 × 10^6^ on the accuracy and precision of δ^18^O­(PO_4_) values using three different isotope standards. Figure S7 shows that these operating parameters affect precision quite substantially, with a reduction of relative standard errors from 3.9 to 0.4‰ as AGC targets increase for the above-mentioned range. By contrast, the accuracies of the δ^18^O­(PO_4_) values exhibit only subtle changes and deviations from the reference data. Consequently, ^18^O/^16^O ratio measurements in this study were performed at an AGC target of 2 × 10^5^. Our findings suggest that increased AGC targets improve ion-counting statistics through higher ion densities in the Orbitrap while not being impaired substantially from coalescence. Similar observations were made by Mueller et al.^5^ who used an AGC target of 10^6^ for a combined C and H isotope analysis of acetate by Orbitrap MS.

Data for different mass resolutions are shown in Figure S8. The relative standard error of ^18^O/^16^O ratio measurements varies substantially with mass resolution. Relative standard errors decreased monotonically with decreasing mass resolution and increasing ion acquisition time (Figure S8a,b), in agreement with the notion of increased ion-counting efficiency at low mass resolution. At data acquisition periods of 15 min, for example, the relative standard error of the measurement decreases from 5.0 to 0.7‰ at mass resolutions of 280,000 and 17,500, respectively. The deviations in raw ^18^O/^16^O ratios (Figure S8c) could be attributed to shorter scan rates, and thus smaller ion counts as well as different decay rates of heavy isotopologues in the Orbitrap mass analyzer, as proposed previously.^5^ As shown in Figure S8d, we observe an increase in δ^18^O­(H_2_PO_4_^–^) from +39.7‰ to +50.6‰ for mass resolutions from 17,500 to 280,000. The values closest to the expected value from EA-IRMS (+41.0‰) are measured at mass resolutions of 70,000 and 140,000 (+39.7 ± 1.2‰ and +42.5 ± 1.7‰, respectively).

The systematic evaluation of Orbitrap MS operating parameters allows for determining accurate ^18^O/^16^O ratios with acquisition errors, which scale with ion acquisition times. Furthermore, our large data set of δ^18^O­(PO_4_) values of laboratory standards acquired over an extended sampling period with identical instrument parameters defined above (e.g., 15 min acquisition time, *R* of 70,000) enables consideration of long-term experimental reproducibility of ^18^O/^16^O ratio measurements. The evaluation of 165 δ^18^O­(PO_4_) values of six laboratory standards from Figure 1a reveals an average standard deviation of ± 2.80‰, with individual standard deviations of triplicate measurements ranging from 0.1 to 5.3‰. This overarching experimental reproducibility pertinent to our procedure for Orbitrap MS is 1 order of magnitude higher than uncertainties of <0.2‰ for ^18^O/^16^O ratio measurements of Ag_3_PO_4_ by EA-IRMS.^22,38^ However, sample sizes for Orbitrap MS are approximately 2 orders of magnitude smaller (9 nmol of H_3_PO_4_ vs 960 nmol (400 μg) of Ag_3_PO_4_).

If used as a measure of total instrument uncertainty,^39^ the experimental uncertainty of δ^18^O­(PO_4_) derived here for Orbitrap MS measurements allows one to assess operational method quantification limits in a manner done for compound-specific isotope analysis by gas and liquid chromatography IRMS.^40^ The applied moving mean procedure is equally based on the fundamental relationship between precision and ion-counting statistics.^41^ It is used to derive the lowest analyte concentration for a given procedure of isotope ratio measurements within uncertainty bounds considered representative for the instrumental approach (e.g., refs 42–44 ). Figure 1b shows δ^18^O­(PO_4_) values of laboratory standard D determined with phosphate concentrations between 1 and 50 μM. Only data acquired from solutions containing 25 and 50 μM were within the limits of an experimental uncertainty of ±2.80‰, suggesting that at least 25 μM of phosphate are required to obtain ^18^O/^16^O ratios with acceptable precision. This outcome suggests that a moving mean procedure can be applied to define operational method quantification limits (MQLs) for isotope ratio measurements by Orbitrap MS, provided that instrument operating parameters, sample volumes, and sample throughput have been defined.

### δ^18^O from PO_3_^–^-Fragments of Phosphate and Organophosphorus Compounds

Following previous findings for ^18^O/^16^O ratio analysis of phosphate from PO_3_^–^ fragment isotopologues instead of those from H_2_PO_4_^–^,^23^ we evaluated in-source collision-induced dissociation (CID) energies and high-energy collisional dissociation (HCD) for the accuracy of δ^18^O­(PO_4_) of phosphate and two organophosphonates (aminomethyl phosphonic acid, AMPA, and 2-aminoethyl phosphonic acid, AEPA). All other instrument operating parameters were identical to those described in the previous section.

Figure 1c shows the relative abundance of the light (^16^O) isotopologue fragments of H_2_PO_4_^–^, PO_3_^–^, and PO_2_^–^ vs in-source CID energy. δ^18^O­(PO_3_^–^) values for PO_3_^–^ fragments were obtained at an in-source CID energy of 45 eV, where PO_3_^–^ fragment intensities exceeded 90% abundance. δ^18^O­(PO_3_^–^) was calculated from ^18^ *R* with the ratios of ^31^P^18^O^16^O_2_^–^ (*m*/*z* = 80.964) and ^31^P^16^O_3_^–^ (*m*/*z* = 78.959) isotopologues. In Figure 1d, the O isotope signatures of the six laboratory phosphate standards determined without fragmentation, δ^18^O­(H_2_PO_4_^–^), are correlated against those of the identical standards after fragmentation at 45 eV, δ^18^O­(PO_3_^–^), for the two sample introduction techniques and three measurement campaigns. Measurements in the direct-infusion mode show a slight underestimation of δ^18^O­(PO_4_) from PO_3_^–^ fragments by 2.4% from the linear correlation slope over a range of 90‰ and an increased deviation at δ^18^O values ≥40‰. An equally minor overestimation of the number of O isotope signatures (3.8%) was obtained with the flow-injection mode. Regression slopes and y-intercepts are provided in the caption of Figure 1d. Identical results are obtained when δ^18^O­(PO_3_^–^) values are correlated with δ^18^O­(PO_4_) reference data from EA-IRMS (Figure S9) with even better agreement (slope of 1.009). These observations imply that no conceivable and systematic O isotope fractionation in phosphate occurs through CID. Contrary to previous findings by Wang et al.,^23^ who used a different type of Orbitrap MS instrument, we do not observe an improvement of either the accuracy or precision of ^18^O/^16^O ratio measurements in phosphate after in-source fragmentation. The good agreement of δ^18^O­(PO_4_) obtained with and without in-source fragmentation with the proposed instrument operating parameters enables us to probe for ^18^O/^16^O ratios in organophosphorus compounds.

The results of the ^18^O/^16^O ratio measurements of PO_3_^–^ isotopologues from two aminoalkyl phosphonates are shown in Figures 1e,f, S10, and S11. Following the same approach, δ^18^O­(PO_3_^–^) of AEPA was determined at in-source CID energies where PO_3_^–^ ions were the most abundant (60 and 70 eV). Even though the share of intact AEPA remains at 25–30% at these collision energies, δ^18^O­(PO_3_^–^) of AEPA (−4.21 and −5.34‰ at 60 and 70 eV, respectively) agreed within uncertainty with data from analysis by EA-IRMS (−3.87 ± 1.64‰, Figure 1f). By contrast, the fragmentation of AMPA gave rise to very similar relative abundances of fragments PO_3_^–^ and PO_2_^–^ over the CID range of 20–90 eV. None of these fragments exceeded 50% relative abundance (Figure S11). δ^18^O­(PO_3_^–^) at low CID energies of 25 to 35 eV with residual AMPA fractions of 44–65% deviated by up to −52‰ from the EA-IRMS-based reference value of +13.8‰. This large deviation points to substantial isotope fractionation of AMPA during the in-source CID fragmentation process.

Results from fragmentation experiments with phosphate, AEPA, and AMPA using the HCD cell are shown in Figures S12–S14. We observe an identical outcome for the accuracy of δ^18^O­(PO_4_) for the analysis of phosphate solutions as with in-source CID fragmentation. After choosing the (normalized) collision energies leading to the highest relative abundance of PO_3_^–^ fragments, δ^18^O­(PO_3_^–^) correlated linearly with δ^18^O­(PO_4_) from EA-IRMS measurements of the six H_2_PO_4_^–^ standard solutions spanning a δ^18^O­(PO_4_)-range of 90‰ (Figure S12). As observed in two measurement campaigns, correlation slopes varied between 0.982 ± 0.033 and 1.003 ± 0.030 but were not significantly different from unity. Data for the two aminoalkyl sulfonates, however, revealed substantial isotope fractionation associated with the fragmentation process (Figures S13 and S14). While PO_3_^–^ abundance exceeded 97% from AEPA fragmentation at optimized collision energies (Figure S13a,c), no such regime was found for AMPA. Regardless of the collision energy, δ^18^O­(PO_3_^–^) determined at maximum PO_3_^–^ abundance deviated by up to 49 and 75‰ for AEPA and AMPA, respectively. This observation contrasts the data for CID fragmentation experiments which, at least, allowed for the accurate determination of ^18^O/^16^O ratios in AEPA. Further work is required to identify optimized HCD settings for accurate O isotopologue analysis in fragments of organophosphorus compounds.

### Effects of Aqueous Matrix and Cosolutes

By selection of appropriate instrument operating parameter settings, the isotopic analysis of the relevant H_2_PO_4_^–^ isotopologues in the Orbitrap mass analyzer becomes possible. We tested the hypothesis that other species in the sample matrix outside the selected mass range, primarily water and other oxyanions, can affect the accuracy and precision of ^18^O/^16^O ratio measurements through interference with phosphate ionization in the ESI source. Figure 2a shows δ^18^O­(H_2_PO_4_^–^) values of three laboratory standards versus increasing volumetric water content of the methanolic solutions. We find that the δ^18^O­(H_2_PO_4_^–^) values of two of the three standards are within the overall experimental uncertainty of ± 2.80‰ for water contents ≤50 vol %. Standard D exceeds this accuracy limit at 25 vol % water content. Moreover, the precision of ^18^O/^16^O ratio measurements also becomes poor beyond the 50 vol % water-content threshold. We further evaluated the possible O atom exchange in the ESI source by conducting ^18^O/^16^O ratio measurements of phosphate in 5 vol % H_2_O/MeOH mixtures in which the water contained 0.2 to 78.2 vol % of H_2_^18^O. As shown in Figure S15, δ^18^O­(H_2_PO_4_^–^) values remained unchanged within uncertainty limits, suggesting the absence of significant O atom exchange. Our observations imply that the water content in methanolic solutions is critical for measurement accuracy and that aqueous samples require at least a 2-fold dilution with methanol prior to the O isotope analysis.

**Figure 2:**
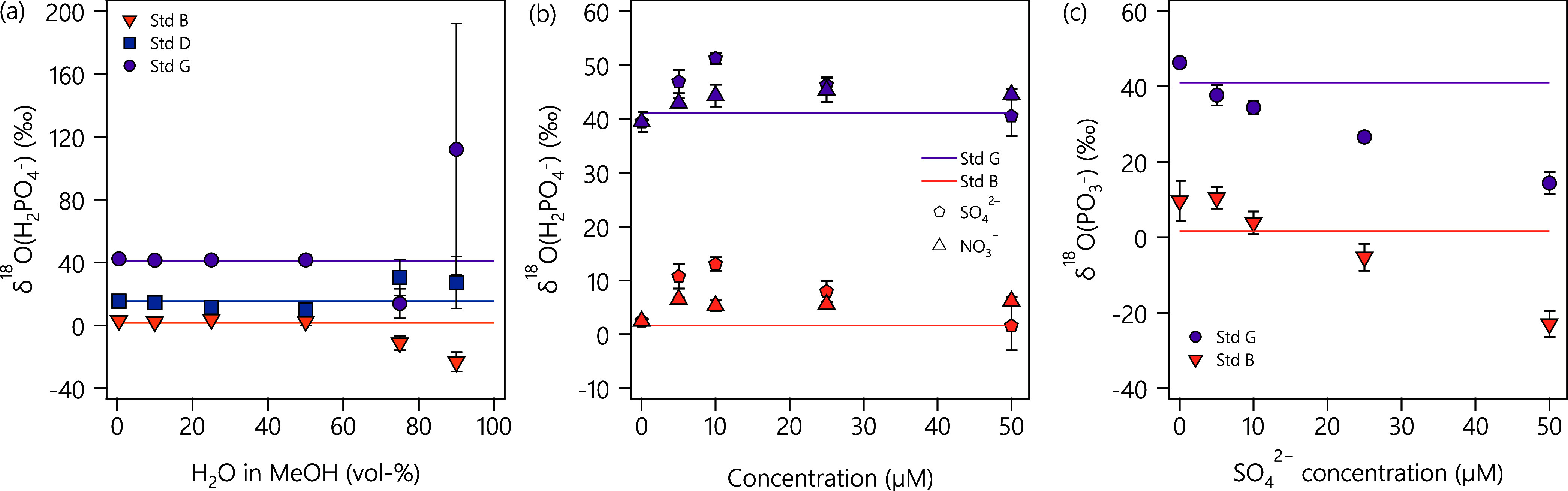
(a) Effect of increased water content in MeOH (vol %) on the precision and accuracy of the O isotope ratio measurement. δ^18^O­(PO_4_) values in samples with a water content up to 50 vol % in MeOH are accurate and precise. The solid line shows reference values from EA-IRMS. Samples containing 75 and 90 vol % of water show an offset from expected values and exhibit larger errors. (b) Effect of nitrate and sulfate concentrations in phosphate samples on the accuracy and precision of δ^18^O­(PO_4_). With increasing nitrate or sulfate concentration, the accuracy and precision of the δ^18^O­(H_2_PO_4_^–^) decrease. (c) δ^18^O from fragmentation of PO_4_^3–^ to PO_3_^2–^ in the samples containing sulfate.

The effect of nitrate and sulfate concentrations up to 50 μM on the determination of δ^18^O­(PO_4_) without and with phosphate fragmentation as δ^18^O­(H_2_PO_4_^–^) and δ^18^O­(PO_3_^–^) values is shown in Figure 2b,c, respectively. Isotope signatures generally deviate from δ^18^O­(PO_4_) of the isotope standards used. δ^18^O­(H_2_PO_4_^–^) values transiently increase with increasing concentrations of both nitrate and sulfate. In the presence of nitrate, we also observe a decrease in the total ion current in the mass range 95–105 Da, suggesting interference of nitrate with H_2_PO_4_^–^ ionization. This effect would be consistent with the notion that oxyanions with lower p*K*_a_ of the conjugate acid exhibit higher ionization efficiencies.^45,46^ The opposite effect on the total ion current is found in the presence of sulfate, given that HSO_4_^–^ ions fall within the chosen quadrupole mass range (Figure S16). Evidence for interference of sulfate during ionization was obtained indirectly from in-source CID fragmentation of phosphate. Under these conditions, we find a monotonic decrease of δ^18^O­(PO_3_^–^) by more than 20‰ with an increasing sulfate concentration (Figure 2c). This observation contrasts conclusions made by Wang et al.,^23^ who claim to avoid HSO_4_^–^ interferences on ^18^O/^16^O ratio measurements through phosphate fragmentation. While this interpretation is valid for isobaric interferences, our data suggest that the observed offset of δ^18^O­(PO_3_^–^) values from those of EA-IRMS measurements could also stem from artifacts pertinent to the phosphate ionization process in the ESI source. Collectively, the available data on oxyanion interferences during ^18^O/^16^O ratio measurements of phosphate strongly suggest that such ions be removed prior to analysis by Orbitrap MS.

### Sample Purification Procedure on the Basis of Selective Interactions of Phosphate with Zr-Based Metal–Organic Frameworks

To eliminate other oxyanions from the samples, we developed a procedure for the selective extraction of phosphate with Zr-based MOFs. The extraction is followed by the recovery of phosphate from the sorbent material and a cleanup step to obtain aliquots with low cation concentrations. A schematic representation of the procedural steps from phosphate extraction to ^18^O/^16^O ratio measurements is shown in Figure 3, and a detailed description of the procedure is given in Section S2.6.1. Typical sample sizes, molar masses, and concentrations of the relevant species are summarized in Table S5. We evaluated the feasibility of the various steps of the proposed approach for the processing of 300 nmol of phosphate to a final PO_4_^3–^ concentration of ≥50 μM that is required for accurate ^18^O/^16^O ratio measurements by Orbitrap MS. Finally, we applied the optimized procedure on two sample sets to confirm the removal of both interfering oxyanions (sulfate, nitrate) while maintaining the accuracy of δ^18^O­(PO_4_) values.

**Figure 3:**
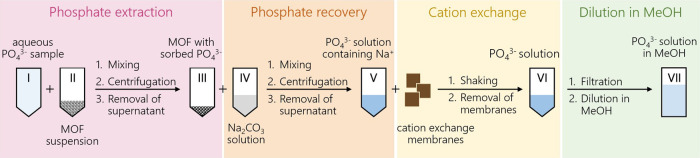
Four-step phosphate extraction and purification procedure using MOFs to obtain a methanolic solution for Orbitrap MS analysis from an aqueous sample. The principal steps include (1) phosphate extraction, (2) phosphate recovery, (3) cation exchange, and (4) dilution in methanol to ensure compatibility of sample matrices with ESI-Obitrap MS analysis.

First, we verified the Langmuir-type sorption behavior of phosphate at Zr-based MOFs observed previously^26^ with experiments at moderately low total sorbent and sorbate concentrations (≤50 mg/L Zr-MOF). The results confirm fast removal of phosphate from aqueous solutions within minutes and sorption capacities in the mmol/g-range, as demonstrated earlier by others.^26^ Figure S17 illustrates the same qualitative sorption behavior applied under experimental conditions used for quantitative extraction of up to 900 nmol of phosphate for O isotope analysis from suspensions containing 380 mg/L of Zr-MOF. On that basis, we mixed 750 μL of a KH_2_PO_4_ standard solution (solution I in Figure 3) with 750 μL of a Zr-MOF suspension (760 mg/L, denoted as suspension II), followed by shaking overnight on an overhead shaker. The suspension (1.5 mL) was then centrifuged (2655 rcf, 15 min, 25 °C), followed by removal of 1.4 mL of the supernatant solution with a pipet. Phosphate concentrations in the supernatant were determined with a colorimetric assay (Section S1.3.1).

Phosphate recovery from the Zr-MOFs was achieved through the anion exchange of phosphate for carbonate. We note that MOF disintegration is possible at high pH,^29^ which might be problematic if sorbents are to be reused. Here, 1 mL of Na_2_CO_3_ solution (IV of varying concentrations, 80–340 mM) was added to 100 μL of the MOF-slurry (III) and shaken for up to 11 days. The effect of Na_2_CO_3_ concentration in solution IV on phosphate recovery is shown in Figure S18 in terms of the ratio of the molar excess of carbonate over phosphate. Phosphate recoveries that exceeded 75% required at least 1000-fold excess of carbonate. After 1.1 mL suspension was centrifuged (conditions see above), 1 mL of the phosphate-containing supernatant was removed (solution V). Solution V contained up to 680 μmol of sodium ions (approximate concentration: 680 mM).

Given that elevated cation concentrations can potentially compromise concentration and isotope ratio analyses of Orbitrap mass spectrometers, supernatant V was subject to cation exchange. We used cation exchange membranes of 1 cm^2^ conditioned in a 2 M nitric acid solution for 24 h prior to usage with sodium absorption capacities of 41 ± 12 μmol of Na^+^ per resin sheet (Table S7). Depending on the carbonate concentration in solution V, up to 17 cation-exchange cycles were necessary, each taking 15 min to equilibrate (a total processing time of up to 4 h). Finally, the aliquots were filtered with a 0.2 μm syringe filter before being diluted with at least identical amounts of MeOH to obtain H_2_O contents ≤50 vol %. The final phosphate concentration in solution VII was 50 μM.

Following this procedure, we generated two sets of samples for ^18^O/^16^O ratio measurements of phosphate. Sample set 1 consisted of aqueous solutions of NaH_2_PO_4_ and was used to study the consequences of variable PO_4_^3–^ recovery and equilibration times. While the conditions for phosphate extraction were kept constant (i.e., 24 h for the extraction step), we varied the carbonate/phosphate ratios in the desorption step and the time for equilibrating mixtures of solutions III and IV over three different time periods (1, 6, and 11 days). Figure 4 shows that extended time periods for phosphate desorption exceeding 6 days in carbonate-containing MOF suspensions and high phosphate recoveries enable accurate ^18^O/^16^O ratio measurements. δ^18^O­(PO_4_) values after 6 days of equilibration matched within experimental uncertainty those of the aqueous solutions of NaH_2_PO_4_ (+ 23.9 ± 2.8‰) that had not undergone the sample preparation procedure. Short equilibration for only 1 day led to PO_4_^3–^ becoming enriched in ^18^O by up to +10‰ regardless of the relative phosphate recovery. This observation suggests a kinetically driven fractionation of the O isotope that leads to a preferential desorption of ^18^O-containing isotopologues of phosphate from Zr-BDC-MOFs. Sample sets after 1 and 11 days of equilibration show variations of δ^18^O­(PO_4_) values over approximately 5‰ that exceed the experimental uncertainty. This phenomenon may be related to effects of the sample matrix from the phosphate extraction procedure (e.g., high carbonate concentrations in solutions VI and VII) on phosphate ionization by ESI, which warrants further study.

**Figure 4:**
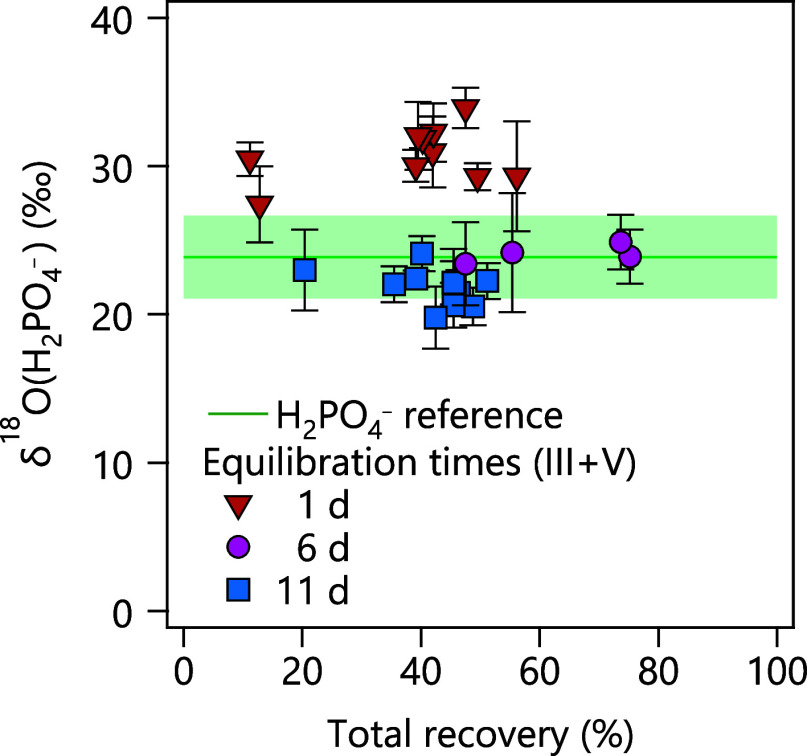
δ^18^O­(H_2_PO_4_^–^) values for the MOF extraction and purification procedure with different equilibration times after mixing suspension III and solution IV (see text). The green line and shaded area show the expected δ^18^O­(H_2_PO_4_^–^) value with an uncertainty band of ±2.8‰.

Finally, we used sample set 2 to test the applicability of the procedure for removing interfering anions, specifically sulfate and nitrate, which were shown to cause deviations from accurate ^18^O/^16^O ratio measurements in phosphate (Figure 2). To that end, we processed a sample containing 660, 150, and 850 nmol of phosphate, nitrate, and sulfate, respectively, in a total volume of 750 μL according to the procedure outlined in Figure 3 and described above. NaH_2_PO_4_, with a known δ^18^O­(PO_4_) value, was used as the phosphate source. Table S6 summarizes the results of oxyanion concentration measurements of phosphate, nitrate, and sulfate in terms of the anion mass removed and retained during sample processing. It should be noted that that phosphate was present in excess of the sorption capacity of Zr-MOF, mimicking a situation of an environmental analysis where the sample cleanup procedure is implemented by adding a predetermined amount of MOFs to withdraw the minimum amount of analyte for ^18^O/^16^O ratio measurements by Orbitrap MS. Our data show that 98 and 95% of the nominal nitrate and sulfate mass, respectively, were detected in solution III, whereas 5 and 8% of the mass were present in solution VI that was subject to O isotope analysis. This solution also contained 30% of the original phosphate. The δ^18^O­(H_2_PO_4_^–^) value of these samples was +25.3 ± 3.0‰ (Table S6) and thus agreed within uncertainty to its reference value of +23.9 ± 2.8‰ (Figure 4).

## Conclusions

ESI-Orbitrap MS offers promising avenues to complement the analysis of ^18^O/^16^O ratios in phosphate and organophosphorus compounds in aqueous solutions by EA-IRMS through smaller analyte sizes, the ability to evaluate molecular fragments selectively, and improved instrument availability. The novel approach is thus likely to promote the use of inorganic phosphate as well as organic compounds as with phosphate and phosphonate moieties as isotopic markers for the study of metabolic processes or pollutant degradation and formation dynamics. Our work illustrates the role of various Orbitrap MS operating parameters for the long-term accuracy and precision of such measurements of phosphate as well as PO_3_^–^-fragments from organophosphorus compounds. The excellent agreement of δ^18^O­(H_2_PO_4_^–^) and δ^18^O­(PO_3_^–^) shown here indeed offers interesting perspectives to extend the ^18^O/^16^O ratio analysis to structurally more complex compounds on the basis of collisional induced dissociation, using both in-source CID- and HCD-based^23^ fragmentation. However, to benefit from the versatility of Orbitrap MS for isotopic analysis, the effects of the sample matrix on the ionization of the target analyte in the ESI source require further and systematic study.

We illustrate that an analyte purification procedure with an adequate selectivity for phosphate can be established successfully. While such a procedure still requires several steps prior to the introduction of methanolic solutions into the Orbitrap MS, it is less time- and labor-intensive for aqueous samples than already established procedures for IRMS. However, this advantage currently comes at the expense of lower precision (i.e., ±2.8‰ for Orbitrap MS vs ≈ ±0.2‰ for EA-IRMS^21,22^ ) which we quantified here through measurements over an extended time period. The proposed approach is thus suited for analyses of samples from laboratory experiments, which offer some control over the sample matrix composition. Promising examples include studies of enzymatic phosphoryl transfer reactions, where the measurement of ^18^O/^16^O ratios from phosphate and organophosphorus compounds will allow the isotopic characterization of both reactants or products of such processes. Future work needs to address how sample purification procedures can be improved and extended to enable enrichment of small phosphate concentrations from larger water volumes while maintaining the selectivity for phosphate and minimizing the subsequent ion removal steps prior to isotopic analysis.

## Supplementary Material



## Data Availability

The raw data underlying the findings of this study are available at 10.25678/000EPT.

## Notes

Chemicals used, synthesis and characterization of MOFs, analytical methods for oxyanion quantification, additional results for long-term reproducibility, influence of instrument parameters on ^18^O/^16^O ratio measurement accuracy, fragmentation of organophosphates and ^18^O/^16^O ratios from PO_3_^2–^, documentation of effects of oxygen atom exchange, aqueous matrix and cosolutes on δ^18^O­(PO_4_), properties of solutions and suspensions used in the sample purification procedure, and phosphate sorption isotherms for Zr-MOF (PDF)
