# Supplementary material for: Oxygen Isotope Analyses of Phosphate and Organophosphorus Compounds by Electrospray Ionization Orbitrap Mass Spectrometry
Source: Anal Chem. 2025 Oct 10;97(41):22777–86. doi: 10.1021/acs.analchem.5c04367 (PMC12547861; doi:10.1021/acs.analchem.5c04367)
Supplement: Supplementary file 1 [file ac5c04367_si_001.pdf]

Supporting Information

Oxygen Isotope Analyses of Phosphate and Organophosphorus Compounds by Electrospray Ionization Orbitrap Mass Spectrometry

Nora M. Bernet,[1,2] Cleo Soldini,[1] Timo M. O. Felder,[3] Kristýna Lapčíková,[4] Cajetan Neubauer,[5] Wendy L. Queen,[3] Ralf Kaegi,[1] Federica Tamburini,[6] and Thomas B. Hofstetter[1,2*]

[1]Eawag: Swiss Federal Institute of Aquatic Science and Technology, 8600 Dübendorf, Switzerland, [2]Institute of Biogeochemistry and Pollutant Dynamics (IBP), ETH Zürich, 8092 Zürich, Switzerland; [3]Institute of Chemical Science and Engineering (ISIC), École Polytechnique Fédérale de Lausanne (EPFL), 1951 Sion, Switzerland; [4]University of Chemistry and Technology, Prague (UCT Prague), Prague, Czech Republic; [5]University of Colorado Boulder, Boulder, CO 830303, USA; [6]Institute of Agricultural Sciences, ETH Zürich, 8315 Lindau, Switzerland

*Corresponding author: thomas.hofstetter@eawag.ch

28 Pages, 18 Figures, 7 Tables

Contents

S1 Experimental Section **3**

S1.1 Chemicals . . . 3

S1.2 Synthesis and Characterisation of Zr-based Metal-Organic Framework . . . 3

S1.3 Chemical Analysis . . . 5

S1.3.1 Phosphate Quantification . . . 5

S1.3.2 Na^+-Quantification . . . 6

S1.3.3 $SO_4{}^{2-}$-Quantification . . . 6

S1.3.4 $NO_3{}^-$-Quantification . . . 7

S1.4 Instrumentation for $^{18}O/^{16}O$ Ratio Measurement in Phosphate . . . 7

S1.4.1 Direct Infusion . . . 7

S1.4.2 Flow injection . . . 7

S1.5 Data Evaluation . . . 7

S1.6 $\delta^{18}O(PO_4)$ Referencing . . . 8

S2 Additional Results **8**

S2.1 Long-Term Accuracy and Precision . . . 8

S2.2 Identification of optimal instrument parameters . . . 13

S2.2.1 Ion Counting Statistics . . . 13

S2.2.2 Quadrupole Mass Range . . . 13

S2.2.3 AGC Target . . . 14

S2.2.4 Resolution . . . 15

S2.3 $^{18}O/^{16}O$ Ratios of Phosphate and Organophosphorus Compounds from $PO_3{}^-$ Fragments . . . 16

S2.4 Effects of Aqueous Matrix and Co-solutes . . . 22

S2.4.1 Oxygen Exchange During Measurement . . . 22

S2.5 Sulfate . . . 23

S2.6 Sample purification procedure for selective phosphate extraction from aqueous solutions with metal-organic frameworks . . . 24

S2.6.1 Sample Purification Procedure . . . 24

S2.6.2 Competing Anions . . . 25

S2.6.3 Extraction . . . 25

S2.6.4 Recovery . . . 27

S2.6.5 Cation Removal . . . 27

S1 Experimental Section

S1.1 Chemicals

Aqueous solutions were prepared in naopure water (18.2 MW · cm, Arium Pro Ultrapure Water System). Methanol (CH_3OH, LC/MS grade, 99.99%) was used to prepare samples for $^{18}O/^{16}O$ ratio analysis by ESI-Orbitrap MS.

Potassium dihydrogen phosphate (KH_2PO_4, Merck, $\geq$ 99.5%) and 98.2 wt-% ^{18}O-labelled water ($H_2{}^{18}O$, Taiyo Nippon Sanso, $\geq$ 99.99%) were used for the preparation of phosphate isotope working standards. AG 50W X8 cation exchange resin (BioRad, analytical grade, 100-200 mesh, hydrogen form) and HiTrap SP HP cation exchange chromatography columns (Cytiva) conditioned in nitric acid (HNO_3, Merck, 70%), were employed for cation exchange. Ammonium hydroxide (NH_4OH, VWR Chemicals, 25%), silver nitrate ($AgNO_3$, VWR Chemicals, 99-100.5%) and ammonium nitrate (NH_4NO_3, VWR Chemicals, $\geq$ 98.0%) were used to prepare phosphate isotope standards for IRMS analysis. $\delta^{18}O(PO_4)$ of phosphate isotope working standards are ranging between -27 to 67‰, see Table S1.

Nitric acid (HNO_3, Merck, 65%), sulfuric acid (H_2SO_4, Merck, 98%) and formic acid (Merck, 98-100%) were used for tests on phosphate ionisation efficiency.

The following chemicals were required for the preparation of the two reagents of malachite green method for quantification of phosphate quantitative concentrations: sulfuric acid (H_2SO_4, Merck, 98%), malachite green (4-[4-(dimethylamino)phenyl](phenyl)methylidene-*N,N*-dimethylcyclohexa-2,5-dien-1-iminium chloride, Merck, $\geq$ 90%), polyvinyl alcohol (Merck, $\geq$ 99%), ammonium heptamolybdate tetrahydrate ($(NH_4)_6Mo_7O_{24} \cdot 4H_2O$, VWR, 81.0-83.0%) and sodium carbonate decahydrate ($Na_2CO_3 \cdot 10H_2O$, Fluka, $\geq$ 99%). Sodium carbonate decahydrate was also employed in experiments with MOFs.

The following chemicals were used for ESI-Orbitrap MS fragmentation experiments: (2-Aminoethyl)phosphonic acid (Sigma Aldrich, 99%), and Glycerol phosphate ($Na_2C_3H_7O_6P\ x\ H_2O$, Sigma, $\geq$ 99%).

Table S1 $\delta^{18}O(PO_4)$ values of our phosphate isotope working standards determined by EA-IRMS.

Standard	δ^{18}O in ‰ vs. VSMOW
A	–353[a]
B	1.62 ± 0.59
C	282[a]
D	15.33 ± 1.68
E	–26.61 ± 4.04
F	27.20 ± 2.36
G	41.02 ± 2.51
H	66.80 ± 2.93

[a] Standard A and C lay outside of calibrated EA-IRMS range. Apart from preparation of standards E to H by mixing, they were not used in this work.

S1.2 Synthesis and Characterisation of Zr-based Metal-Organic Framework

Zr-1,4-benzenedicarboxylate (Zr-BDC, also known as UiO-66) was characterized by powder X-ray diffraction (PXRD), N_2 adsorption measurements, and thermogravimetric analysis (TGA).

PXRD analysis shows that the experimental diffraction pattern of Zr-BDC matches well with the simulated pattern based on the crystal structure of Zr-BDC (CCDC No. 4512072), confirming a phase of pure and crystalline material (Figure S1a). The BET (Brunauer–Emmett–Teller) specific surface of 1500 area m^2/g was calculated based on the N_2 adsorption isotherm of Zr-BDC (Figure S1b).

Figure S2 shows the TGA data of the as-synthesized Zr-BDC, measured under an air atmosphere. It reveals three major weight-loss steps. The first step, occurring at 25-200°C is primarily attributed to the loss of residual solvent molecules and adsorbed water (approx. 15. wt-%). The second weight loss, observed at 200-400°C, is mainly due to the dehydration of the Zr-oxo cluster. The third weight-loss (above 400°C) corresponds to the decomposition of the organic linkers, leaving ZrO_2 as the residue at 800°C. In a next step in order to quantify the content of missing linkers in the structure, we have to make important assumptions.[1] First, we assume that at 400°C, only BDC linkers and fully dehydroxylated clusters are present. Second, we assume that the residue at 800°C is pure ZrO_2. Third, we assume that missing linkers are charge balanced by O^{2-} at 400°C. Based on this assumptions, we get a chemical composition of $Zr_6O_{6+x}(BDC)_{6-x}$ at 400°C, where x are missing linkers that are charge balanced by O^{2-}.[1] Assuming complete decomposition of the organic linkers to H_2O and CO_2 at 800°C, one mole of MOF yields six moles of ZrO_2. Based on this reasoning, the theoretical weight loss can be calculated using the molecular weight (MW) of the MOF as shown in Equations S1 and S2;

$$\mathrm{MW_{MOF}} = \frac{W_{400°C}}{W_{800°C}} \times 6\mathrm{M\,ZrO_2} \tag{S1}$$

When we set $W_{800°C}$ to 100 % and rearrange to $W_{400°C}$ we get:

$$W_{400°C} = \frac{\mathrm{MW_{MOF}}}{6\mathrm{M\,ZrO_2}} \times 100 \tag{S2}$$

The theoretical weight percentage at 400 °C for a ideal (defect-free) Zr-BDC MOF is 220.21%. Using the experimentally obtained value of 190.04%, we can estimate a missing linker content of approximately 1.5 linkers per formula, corresponding to an idealized chemical

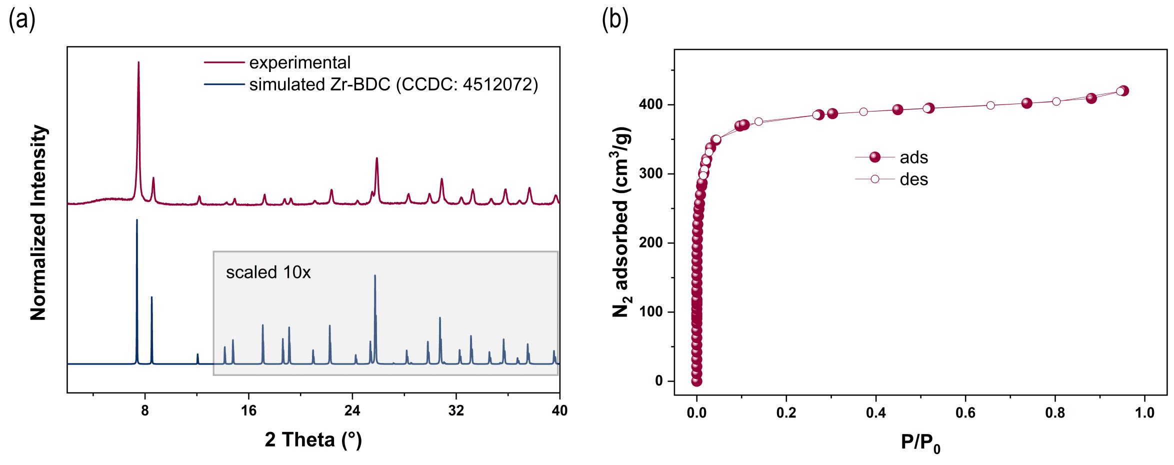

Figure S1 Characterization of Zr-BDC: (a) comparison of experimentally obtained PXRD pattern (red) with calculated pattern (blue) from CIF-file of Zr-BDC (CCDC 4512072), (b) N_2 adsorption isotherm measured at 77 K (for abbreviations see text).

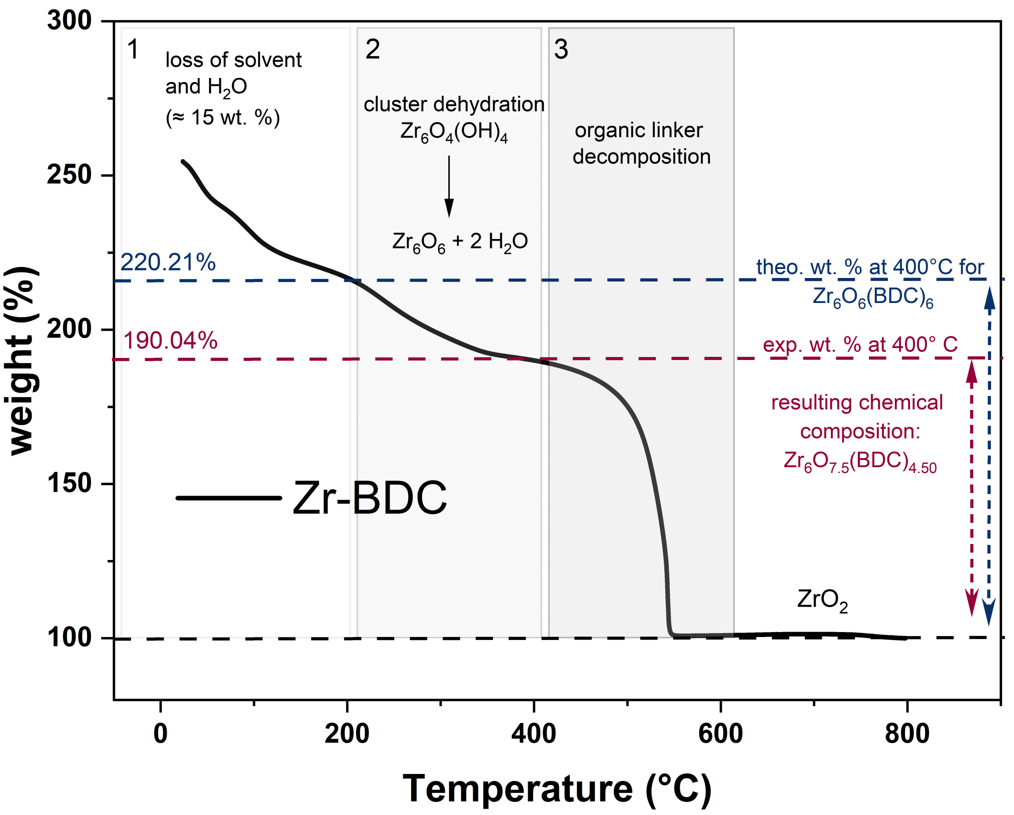

Figure S2 TGA profile of Zr-BDC. The blue and red dashed lines represent the theoretical mass loss for ideal dehydroxylated Zr-BDC (220.21%) and the experimentally observed mass loss (190.04%), respectively.

composition of $Zr_6O_{7.5}(BDC)_{4.5}$ at 400°C.

Size and aggregation behaviour of MOFs were investigated by visual inspection of scanning transmission electron microscopy (STEM, HD2700-Cs, Hitachi, Japan) images recorded at an acceleration voltage of 200 kV (Figure S3). For image formation a secondary electron detector was used.

Stock suspensions of Zr-BDC were prepared in a 0.1 mM sodium chloride solution (NaCl, Merck, $\geq$ 99.5%). Sodium dyhydrogen phosphate anhydrous (NaH_2PO_4, Fluka, $\geq$ 99.0%) was used for testing sample preparation procedures.

S1.3 Chemical Analysis

S1.3.1 Phosphate Quantification

Phosphate was quantified using the malachite green method reported by Ohno and Zibilske[2] using two reagent solutions. Reagent 1 was made by combining an ammonium-molybdate solution (18.08 g of $NH_4Mo_7O_{24}{\cdot}4H_2O$ with 240 mL of nanopure water) and diluted sulfuric acid (168 mL of H_2SO_4 in 500 mL nanopure water). Reagent 2 was prepared by heating 1 L of nanopure water to 80°C and adding 3.5 g of polyvinyl alcohol. After 3 hours of mixing at 80°C and upon complete dissolution of polyvinyl alcohol, 0.35 g of malachite green crystals were added to the solution.

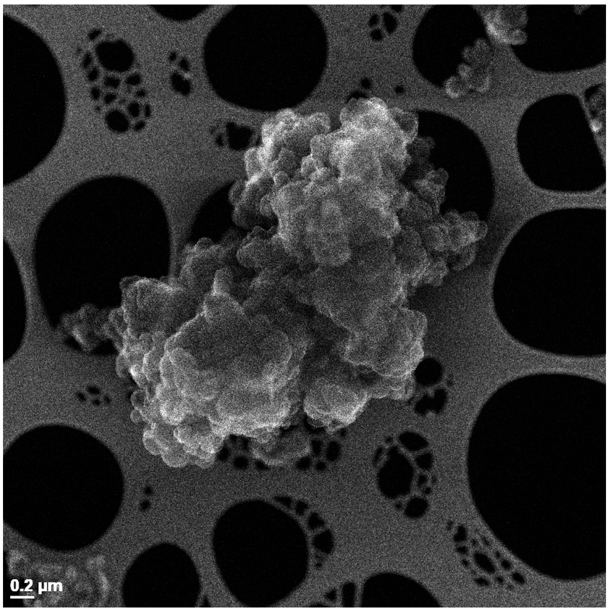

Figure S3 Secondary electron image of Zr-BDC. Image was recorded on a scanning transmission electron microscope.

For phosphate concentration measurements, 1 mL of (diluted) sample solution was mixed with 200 μL of reagent 1. After 15 minutes, 200 μL of reagent 2 were added. After an additional 45 minutes, the solution was placed in UV-Visible spectrophotometer (Jenway, Model 7205) and phosphate concentrations were quantified by measuring the absorption at 610 nm. To that end, a phosphate calibration row was prepared as follows. A phosphate stock solution with a concentration of 323 μM was made by dissolving 10.98 mg of KH_2PO_4 in 250 mL of nanopure water. Seven solutions of 1 mL volume with concentrations between 0.3 to 25.8 μM were obtained by diluting 1 to 80 μL of the stock solution in nanopure water.

S1.3.2 Na^+-Quantification

Sodium (Na^+) concentrations were measured following DIN EN ISO 14911 from the German Institute for Standardization.[3] Prior to concentration measurements, the pH of the aqueous solution was adjusted to a pH between 2 and 3 using a 2 M HNO_3 solution. The samples were then diluted so that their Na^+ concentrations fell into the calibration range of 1-–50 mg Na^+/L. The Na^+ concentration was measured by ion chromatography (Metrohm 930 Compact IC Flex) using a Methrom Metrosep C 6 column (250 mm length/ 4 mm inner diameter) and a conductivity detector.

S1.3.3 ${SO_4}^{2-}$-Quantification

The sulfate (${SO_4}^{2-}$) concentration was measured following DIN EN ISO 10304-1 from the German Institute for Standardization.[4] The ${SO_4}^{2-}$ concentration was measured by ion chromatography (Metrohm 930 Compact IC Flex) with chemical suppression using a Methrom Metrosep A Supp 5 column (100 mm length/ 4 mm inner diameter) and a conductivity detector.

S1.3.4 NO_3^--Quantification

The nitrate (NO_3^-) concentration was measured following DIN EN ISO 10304-1 from the German Institute for Standardization.[4] The NO_3^- concentration was measured by ion chromatography (Metrohm 930 Compact IC Flex) with chemical suppression using a Methrom Metrosep A Supp 5 column (100 mm length/ 4 mm inner diameter) and a conductivity detector.

S1.4 Instrumentation for $^{18}O/^{16}O$ Ratio Measurement in Phosphate

Two common sample introduction methods into the ESI-Orbitrap MS device, namely direct infusion and flow injection, were explored in this work. While direct infusion was selected as most convenient for evaluating the various mass spectrometer parameters and quantifying $^{18}O/^{16}O$ ratios of phosphate in samples which needed quick adjustments, flow injection with an optimised procedure were the method of choice for large sample series. The commonly used sample injection procedures were as follows.

S1.4.1 Direct Infusion

The Q Exactive Plus (Thermo Fisher Scientific) instrument was coupled to a syringe pump (Chemyx, Fusion 100T) that continuously infused the sample at a flow rate of 4 μL/min from a 500 μL glass syringe (Hamilton Robotics) directly into the HESI-II source. Between measurements of different phosphate samples in methanol, the syringe was cleaned with a solution containing 50:50 (v/v) MeOH:H_2O. Samples were analysed for 15 minutes in the mass range from 95 to 105 m/z once a constant total ion current (TIC), TIC variation $\leq$15%, was established.

S1.4.2 Flow injection

The flow injection sample introduction is fully automated with an UltiMate 3000 HPLC system (Thermo Fisher Scientific) coupled to the Q Exactive Plus Orbitrap to allow autonomous sample injections using the HPLC auto-sampler (PAL HTC-xt System). The HPLC loading pump was used to carry eluents of 50:50 (v/v) MeOH:H_2O at a flow rate of 4 μL/min. The 6-port valve of the auto-sampler was equipped with a 100 μL sample loop, resulting in a maximum continuous sample delivery over 28 minutes. The total analysis time per sample was set to 40 minutes to allow 12 minutes of system flushing with the eluent between two measurements and thus to reduce the risk of analyte carryover. The mass spectrometer signals were evaluated in the time range where the TIC was stable resulting in an on average 19 minutes signal. Measurement sequences were set up in alternating blocks of three injections of the reference standard followed by the samples, to improve precision and to allow drift correction (not necessary in our measurement campaigns) over the total analysis time.

S1.5 Data Evaluation

We used the IsoX application (Isotopocule data eXtraction from Orbitrap RAW files; version 2022; distributed via Thermo Fisher Scientific) for extracting relevant ion intensities from RAW files that are generated by the Orbitrap MS control software. isoX files were evaluated in the IsoXL web-based graphical user interface (isoorbi.shinyapps.io/IsoXL, Version 0.53) to obtain $^{18}O/^{16}O$ ratios using the following parameters: Basepeak = M0, Timerange = 0 − 15 min, Segments = 1, remove weak peaks (in %) = 30, remove extreme scans (TICxIT, in %) = 1, and Ratio Method = sum.

S1.6 $\delta^{18}O(PO_4)$ Referencing

We examined the consequences of 1- vs. 2-point calibration of $\delta^{18}O(PO_4)$-values relative to VSMOW reference scale using $^{18}O/^{16}O$ ratio data for standards D, E, and G which cover a range of 68‰ (Table S1). Calculations of $\delta^{18}O(PO_4)$ through 1- and 2-point calibrations are were carried out with eq. 2 (main manuscript) and eq. S3, respectively. Procedures for 2-point calibrations followed recommendations of Gröning[5] and Hilkert et al.[6] which lead to eq. S3.

$$\delta^{18}O(PO_4) = \delta^{18}O_{\text{EA-IRMS, ref1}} + \frac{(\delta^{18}O_{\text{EA-IRMS, ref2}} - \delta^{18}O_{\text{EA-IRMS, ref1}}) \cdot (\delta^{18}O_{\text{raw, smp}} - \delta^{18}O_{\text{raw, ref1}})}{\delta^{18}O_{\text{raw, ref2}} - \delta^{18}O_{\text{raw, ref1}}} \tag{S3}$$

where $\delta^{18}O_{\text{raw},i}$ are values calculated from Orbitrap MS measurements according according to equation 1 and subscript i stands for specimens representing a sample, standard D (ref1), and the second reference sample (ref2, either standard E or G), respectively. $\delta^{18}O_{\text{EA-IRMS}}$ is the $\delta^{18}O(PO_4)$ value of phosphate isotope standards D, E or G quantified by EA-IRMS.

We quantified the apparent accuracy of 1- and 2-point calibrations by examining the slopes of the correlation of $\delta^{18}O(H_2PO_4^-)$ vs. $\delta^{18}O_{\text{EA-IRMS}}$ for 6 laboratory references standards B and D-H (Table S1) in analogy to the evaluations done with data from Figure 1a in the main manuscript. These slopes were calculated from correlations of $\delta^{18}O(H_2PO_4^-)$-values obtained by 1- and 2-point calibrations, respectively. We tested two 2-point calibrations by considering either standard pairs D-E or D-G. The data set for this evaluation originated from four different measurement campaigns run in May 2023, August 2023, July 2024, and Aug 2024. Table S2 lists the correlation equations in the form of eq. S4 where parameters a and b stand for correlation slope and y-intercept, respectively. Δ_{slope} is the deviation of slope a from unity (eq. S5).

$$y = ax + b \tag{S4}$$

$$\Delta_{\text{slope}} = 1 - a \tag{S5}$$

Data from Table S2 show that 1-point calibration exhibited smaller Δ_{slope} values and smaller variations thereof than those obtained from 2-point calibrations, regardless of whether the latter was executed with reference standard pairs D-E or D-G. Average Δ_{slope} (n = 4) of 1- and 2-point calibrations were 0.0093 ± 0.022, -0.032 ± 0.033 (D-E), and 0.0005 ± 0.108 (D-G), respectively. This comparison illustrates that both types of calibrations enable accuracies $< 1\%$ ($\Delta_{\text{slope}} < 0.01$) of $\delta^{18}O_{\text{EA-IRMS}}$-values. However, 2-point calibrations were substantially more variable with a standard deviation of 11% which is almost 5 times as high as the 1-point calibration. For this reason, $\delta^{18}O(H_2PO_4^-)$-values calculated by 1-point calibrations offer the more reliable basis for referencing of $\delta^{18}O(PO_4)$-values from this study. Consequently, all $\delta^{18}O(PO_4)$ values discussed in this study were derived from 1-point calibrations.

S2 Additional Results

S2.1 Long-Term Accuracy and Precision

Figure 1a in the main manuscript shows the $\delta^{18}O(PO_4)$ values of six laboratory working standards determined by direct infusion Orbitrap MS vs. the result of conventional O isotope analysis by EA-IRMS. Table S3 shows those $\delta^{18}O(PO_4)$ values with their standard deviation.

We evaluated the $\delta^{18}O(PO_4)$ of six phosphate laboratory working standards obtained from

Table S2 Correlation equations for comparison of $\delta^{18}O(H_2PO_4^-)$ vs. $\delta^{18}O_{EA\text{-}IRMS}$ of the six laboratory standards for 1- and 2-point calibrations, respectively. Values of slopes a and y-intercepts b from eq. S4 are given with their 95% confidence intervals. Two different 2-point calibrations were evaluated using reference standard pairs D-E and D-G. Δ_{slope} stands for the deviation of correlation slopes from unity (eq. S5).

	1-point calibration		2-point calibration		
Campaign	correlation equation	Δ_{slope}	references	correlation equation	Δ_{slope}
May 23	$y = (1.012 \pm 0.025)x + (0.968 \pm 0.875)$	–0.012	D–E	$y = (1.064 \pm 0.026)x + (0.175 \pm 0.886)$	–0.064
			D–G	$y = (0.939 \pm 0.023)x + (2.095 \pm 0.860)$	0.061
Aug 23	$y = (0.966 \pm 0.028)x + (0.145 \pm 1.037)$	0.034	D–E	$y = (1.034 \pm 0.030)x + (-0.900 \pm 1.055)$	–0.034
			D–G	$y = (0.954 \pm 0.027)x + (0.332 \pm 1.034)$	0.046
July 24	$y = (0.978 \pm 0.034)x + (1.143 \pm 1.160)$	0.022	D–E	$y = (0.986 \pm 0.034)x + (-1.077 \pm 1.197)$	0.014
			D–G	$y = (1.161 \pm 0.040)x + (-3.764 \pm 1.256)$	–0.161
Aug 24	$y = (1.007 \pm 0.030)x + (1.384 \pm 0.974)$	–0.007	D–E	$y = (1.045 \pm 0.031)x + (0.807 \pm 0.983)$	–0.045
			D–G	$y = (0.944 \pm 0.028)x + (2.349 \pm 0.958)$	0.056

Table S3 $\delta^{18}O(PO_4)$ values in ‰ of six laboratory working standards determined by conventional O isotope analysis by EA-IRMS or by direct infusion Orbitrap MS underlaying Figure 1a in the main manuscript. The values were determined over a period of 22 months and 10 measurement campaigns indicated by month and year. Each value consists of the average of at least triplicate measurements with their standard deviation with the exception of measurements in April 2023, which were performed as duplicates and Std D for which we had up to nine single measurements depending on the campaign.

Sample	$\delta^{18}O(PO_4)$ (‰)							
	EA-IRMS	April 2023	May 2023	July 2023	August 2023	May 2024	July 2024	August 2024
Std B	1.62 ± 0.59	–0.17 ± 5.23	3.15 ± 1.36	–0.05 ± 2.18	4.87 ± 1.91	5.25 ± 2.32	3.29 ± 0.95	1.34 ± 0.97
Std D	15.33 ± 1.68	15.33 ± 1.30	15.33 ± 5.47	15.33 ± 1.65	15.33 ± 4.32	15.66 ± 0.54	15.33 ± 1.56	14.12 ± 1.09
Std E	–26.61 ± 4.04	–31.17 ± 2.65	-28.75 ± 1.62	–31.31 ± 2.64	–29.57 ± 1.02	–29.39 ± 1.22	–28.95 ± 2.60	–28.18 ± 5.29
Std F	27.20 ± 2.36	24.48 ± 1.21	24.44 ± 3.17	22.75 ± 2.42	28.93 ± 2.67	26.98 ± 0.85	24.57 ± 2.93	21.77 ± 0.20
Std G	41.02 ± 2.51	37.96 ± 0.14	39.15 ± 0.83	39.60 ± 1.18	10.70 ± 0.29	40.74 ± 1.42	43.75 ± 4.20	39.41 ± 1.09
Std H	66.80 ± 2.93	66.93 ± 0.64	64.76 ± 1.14	67.87 ± 2.14	68.60 ± 1.32	66.36 ± 2.05	65.99 ± 3.11	65.35 ± 2.98

HRMS flow injection measurements to $\delta^{18}O(PO_4)$ values obtained from IRMS measurements, Figure S4. The 1:1 relationship between those values over 6 months demonstrates long-term accuracy and reproducibility of oxygen isotope analysis in phosphate by flow injection HRMS over an environmental relevant range.

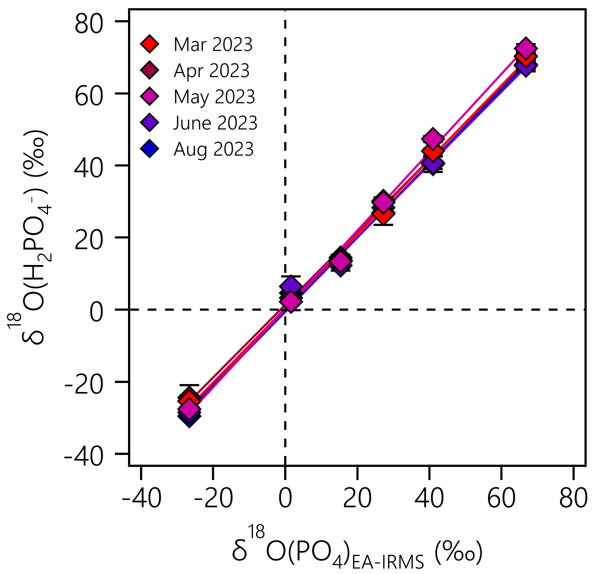

Figure S4 $\delta^{18}O(PO_4)$ values were both measured by EA-IRMS and Orbitrap MS (flow injection) to determine the accuracy and long-term reproducibility of the latter. Comparison of $\delta^{18}O(PO_4)$ measurements by EA-IRMS and Orbitrap MS show an average linear fit of $y = (1.032 \pm 0.032)x + (0.233 \pm 0.709)$.

We investigated the accuracy of isotope ratios measured with flow injection by injecting multiple blocks of standard D and three other standards during a sequence of over 20 hours to identify a possible drift of the system over time. A slight drift can be identified and was corrected by doing a drift correction as described in Hilkert et al.[6], Figure S5. In addition, we tested for memory effect during the analysis and found an average memory effect of 0.16‰ between the different blocks of isotope standards.

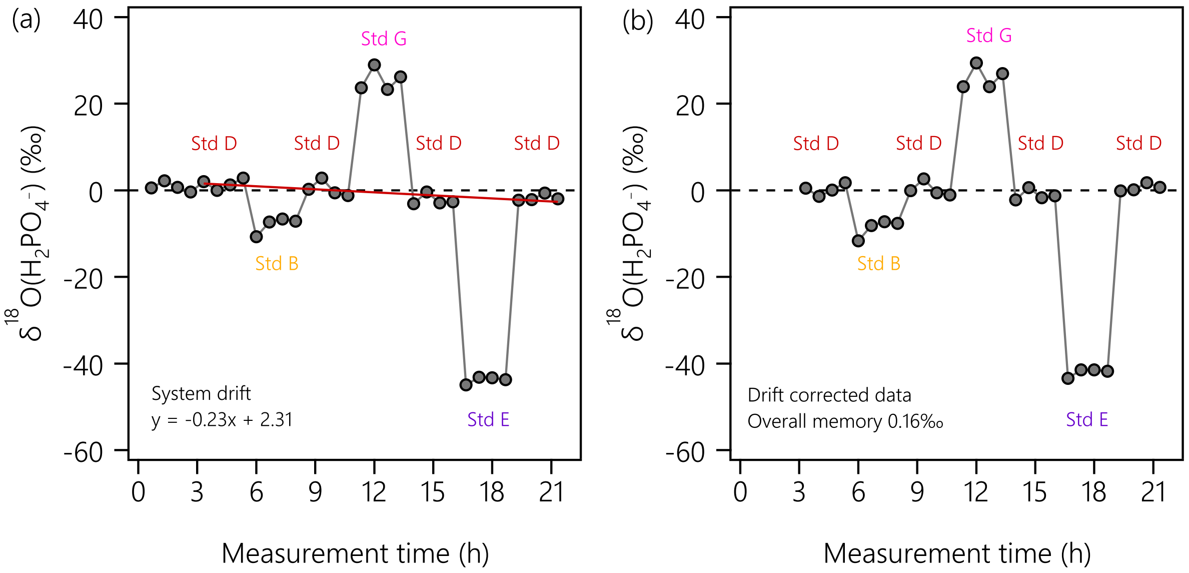

Figure S5 Analysis sequence of phosphate isotope standards alternating between isotopic reference (Std D) and three sample standards (Std B, E and G) to investigate (a) system drift and (b) memory effect (on corrected data) following the procedure used by Hilkert et al.[6] (a) Time courses of δ^{18}O($H_2PO_4^-$) of Std D (calculated from Eq. 1 in main manuscript) illustrate a slight drift of O isotope signatures following a linear trend according to equation δ^{18}O($H_2PO_4^-$) $= (-0.23 \cdot \text{time/h} + 2.31)$‰ over the 22 hours of ^{18}O/^{16}O ratio measurements. The inferred δ^{18}O($H_2PO_4^-$) drift of -0.23‰ per hour is lower compared to -0.43‰ in δ^{15}N of nitrate per hour reported by Hilkert et al.[6] This drift can be corrected and the resulting data is shown in panel (b). Memory effects are calculated from the deviation of δ-value of the first measurement of a measurement block of an isotope standard (typically 4 sample injections) from the average δ-value of the very same block.[6] We find an average memory effect of 0.16 ± 1.85‰ between the different isotope standards blocks, which is slightly higher than the 0.02 ± 0.54‰ observed by Hilkert et al.[6]

S2.2 Identification of optimal instrument parameters

S2.2.1 Ion Counting Statistics

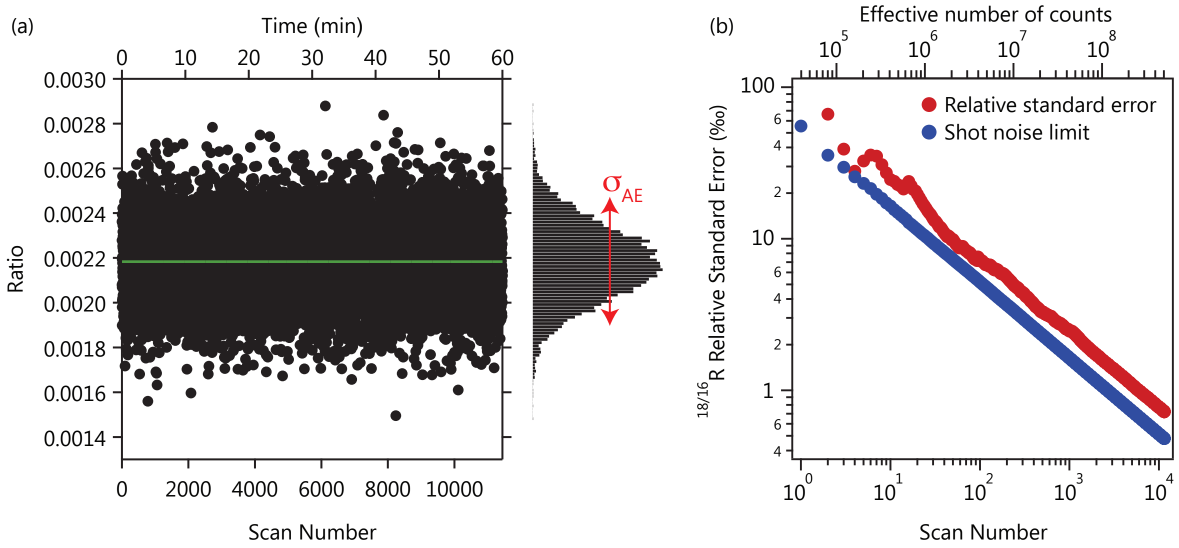

Figure S6 Figure reproduced from Bernet and Hofstetter[7] with permission. (a) $^{18}O/^{16}O$ ratios determined by Orbitrap MS over a 60-minutes acquisition time with continues sample introduction. $^{18}O/^{16}O$ ratio measurements in each scan (black dots) produce a normal distribution around an average value (green line) with σ_{AE} as the acquisition error. (b) Shot noise limit and relative standard error plotted versus the effective number of ion counts during the same 60-minutes acquisition time. The acquisition error of $^{18}O/^{16}O$ ratio measurements decreases equivalent to the theoretical limit imposed by counting statistics.

S2.2.2 Quadrupole Mass Range

Table S4 Effect of different quadrupole ranges on the relative standard error of $^{18}R(PO_4^{3-})$ measurements and the measured $^{18}R(PO_4^{3-})$ values with the corresponding standard deviation.

Quadrupole range (m/z)	Relative standard error (‰)	$^{18}O/^{16}O$ ratio (-)
90 - 110	1.425 ± 0.021	0.008099 ± 0.000027
95 - 105	1.392 ± 0.018	0.008625 ± 0.000014
96 - 102	1.411 ± 0.076	0.008961 ± 0.000025
95.5 - 99.5	1.837 ± 0.083	0.007929 ± 0.000122

S2.2.3 AGC Target

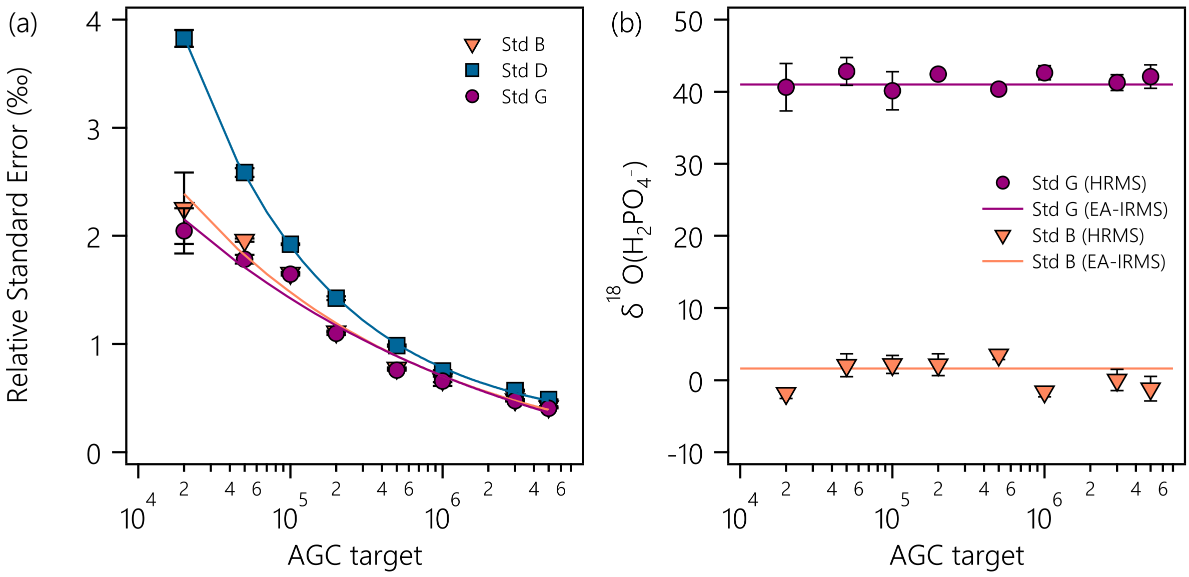

Figure S7 (a) Measurement precision of three different laboratory working standards at different AGC targets. With increasing AGC target, and thus increasing number of ions collected for each scan, the relative standard error of the measured ratios decreases. (b) To consider the measurement accuracy, $\delta^{18}O(PO_4)$ of two isotope laboratory working standards at different AGC targets (dots) were compared to the corresponding expected value from EA- IRMS measurements. When considering both accuracy and precision, we found the best AGC target for oxygen isotope measurements in phosphate to be at 200'000.

S2.2.4 Resolution

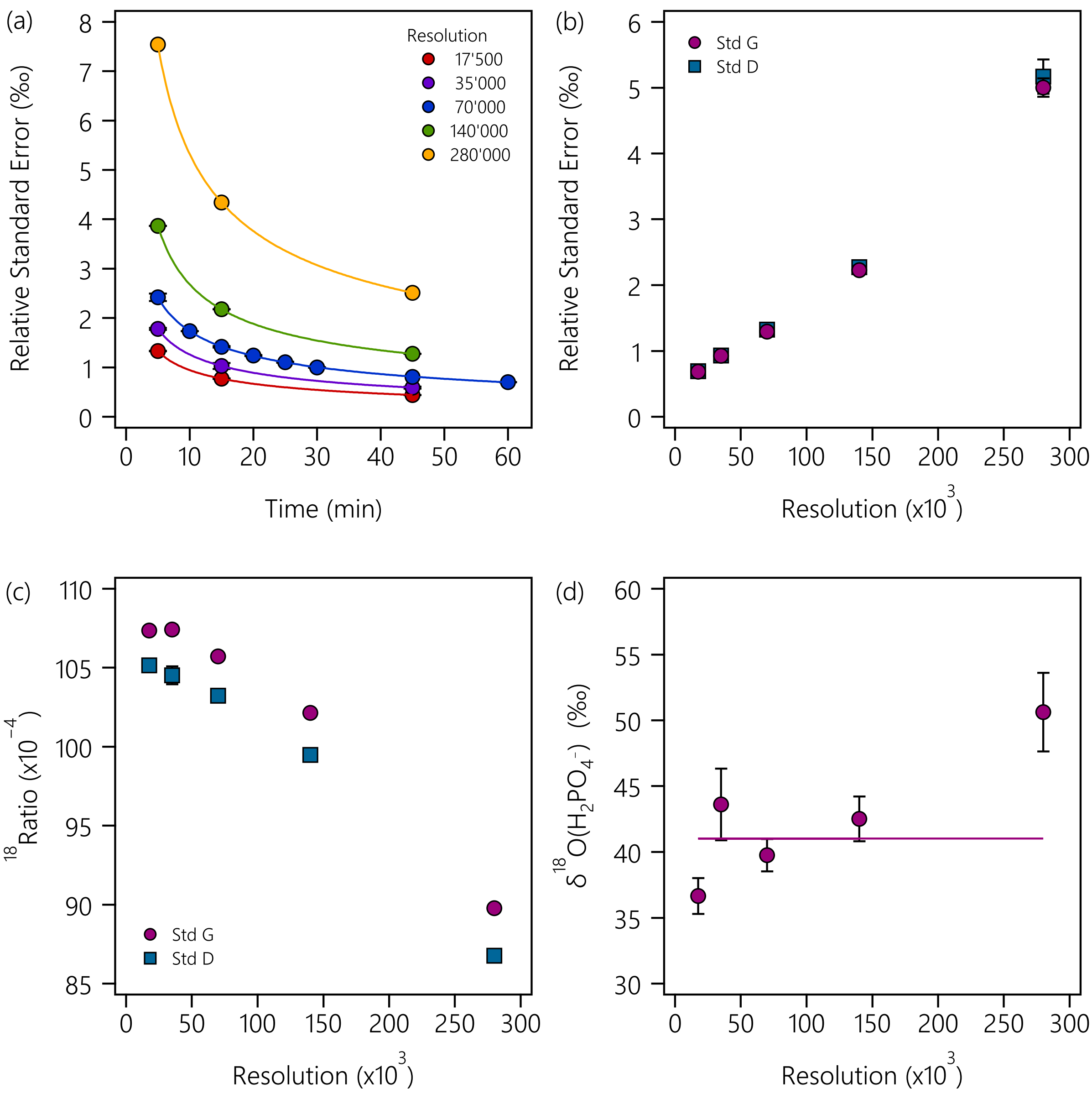

Figure S8 Measurement of phosphate standard solutions at different resolutions for different measurement times ranging from 5 up to 60 minutes. (a) Relative standard error of $^{18}R(PO_4^{3-})$ decreases with increasing measurement time and illustrates that the higher the resolution, the fewer scan per time can be performed. (b) Relative standard errors of laboratory standards D and G at different resolutions. (c) ^{18}O ratios of 15 minutes measurements, (d) $\delta^{18}O(H_2PO_4^-)$ calculated for standard G from measurements show in panel (c).

S2.3 $^{18}O/^{16}O$ Ratios of Phosphate and Organophosphorus Compounds from PO_3^- Fragments

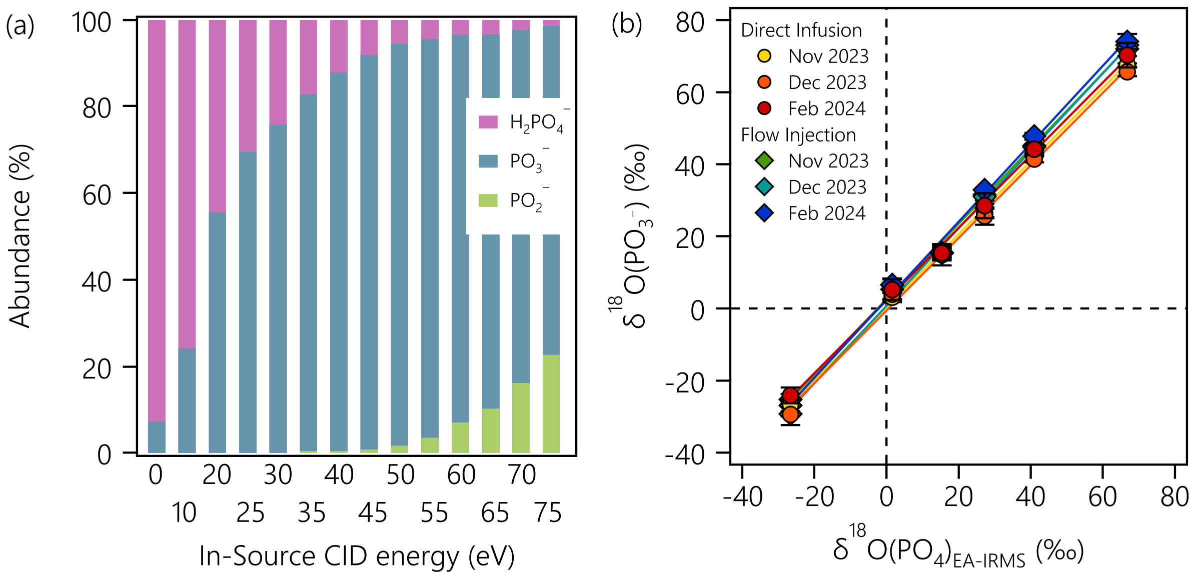

Figure S9 (a) Relative abundances of $H_2PO_4^-$, PO_3^- and PO_2^- at different in-source CID energies. (b) δ^{18}O values determined in PO_3^- fragments from phosphate solutions vs. their δ^{18}O values measured by EA-IRMS. Average linear fit of for measurements performed in direct infusion mode were $y = (1.009 \pm 0.003)x + (0.634 \pm 1.415)$. The corresponding analysis for measurements performed in flow injection mode was $y = (1.067 \pm 0.019)x + (1.889 \pm 1.018)$.

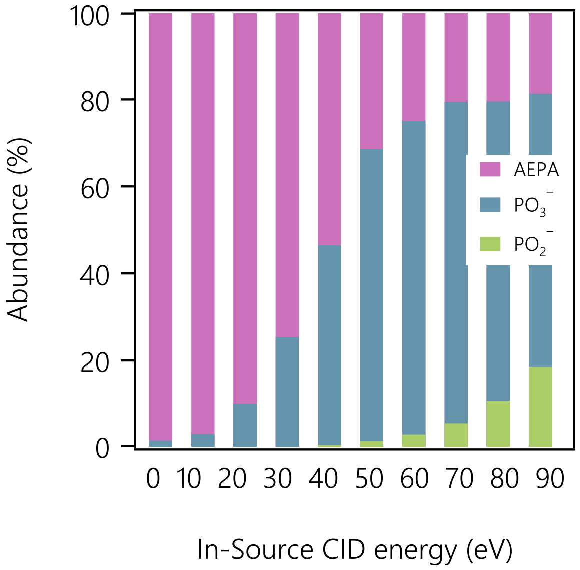

Figure S10 Relative abundances of (2-aminoethyl)phosphonic acid (AEPA) and the two fragments PO_3^- and PO_2^- ions at different in-source CID energies applied to fragment AEPA.

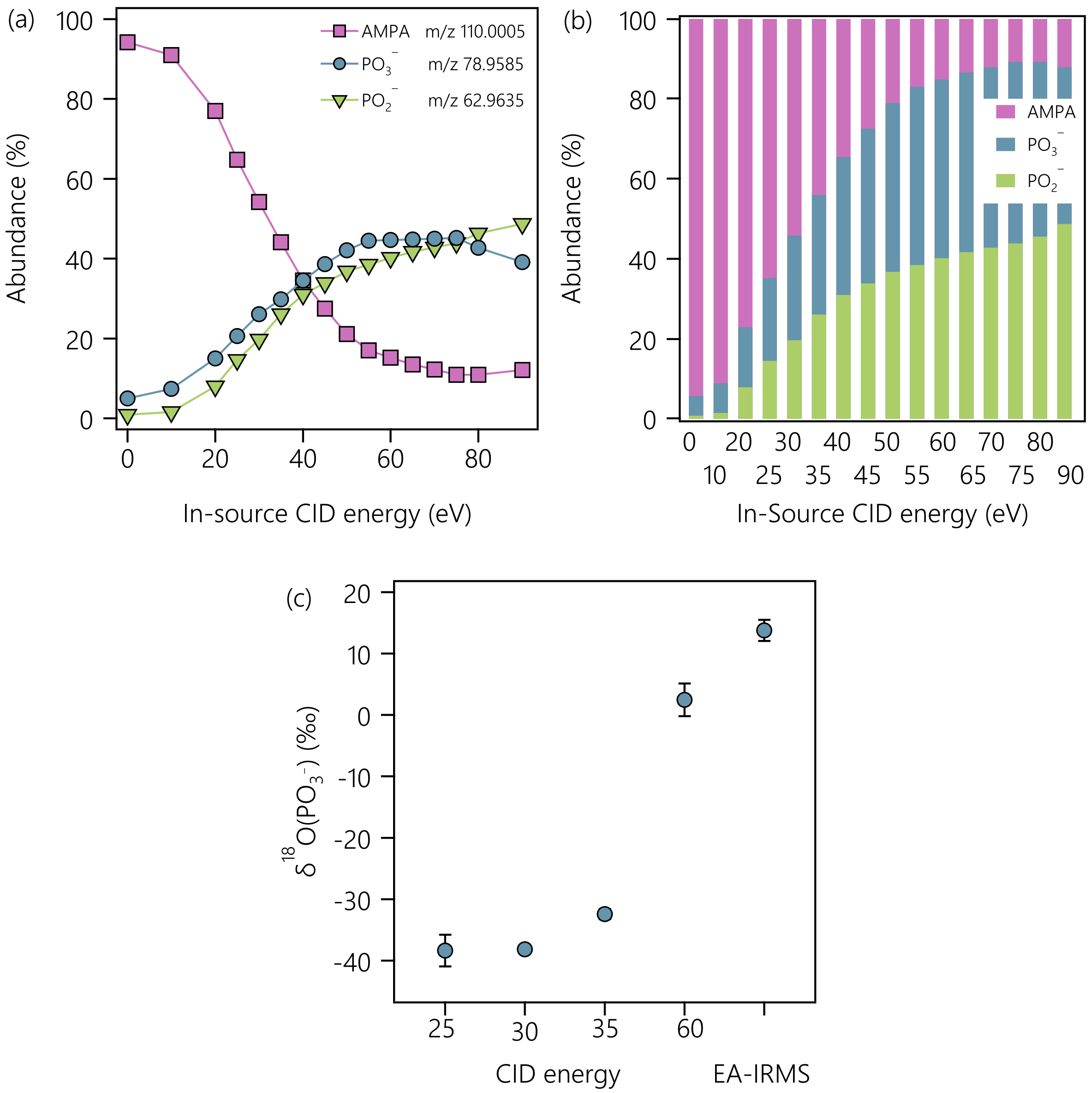

Figure S11 (a) and (b) Relative abundances of aminomethylphosphonic acid (AMPA) ions and its two phosphonate fragments PO_3^- and PO_2^- at different in-source CID energies. (c) $\delta^{18}O(PO_3^-)$ determined at different CID energies (eV).

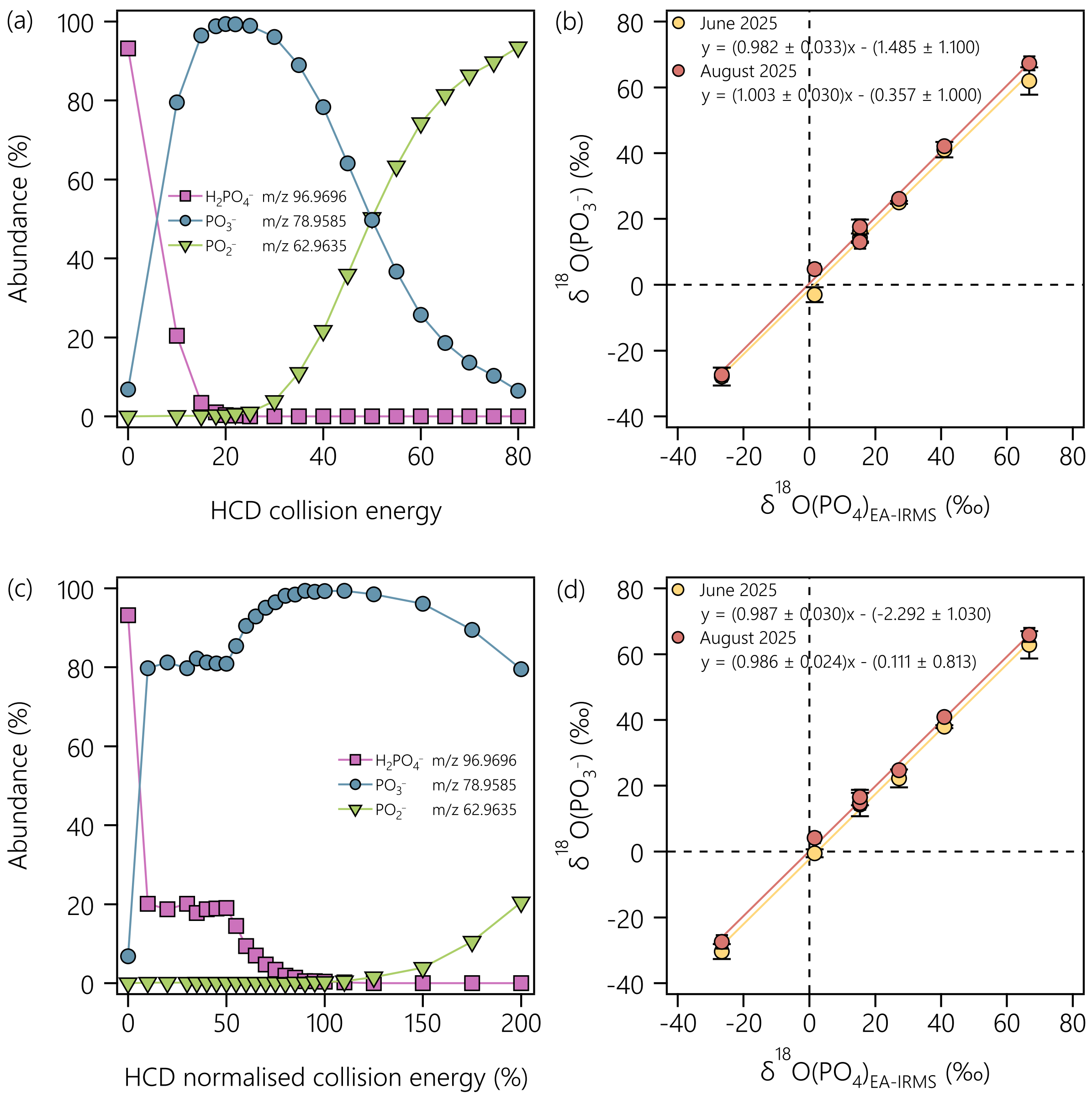

Figure S12 Relative abundances of H_2PO_4 ions and its two phosphonate fragments PO_3^- and PO_2^- at different (a) collision energy (CE) and (c) normalised collision energy (NCE) energies in the higher-energy collision dissociation (HCD) cell. $\delta^{18}O(PO_3^-)$ values of six laboratory working standards determined by direct infusion Orbitrap MS at the following energies (b) CE = 20 eV and (d) NCE = 90% vs. the corresponding values from EA-IRMS.

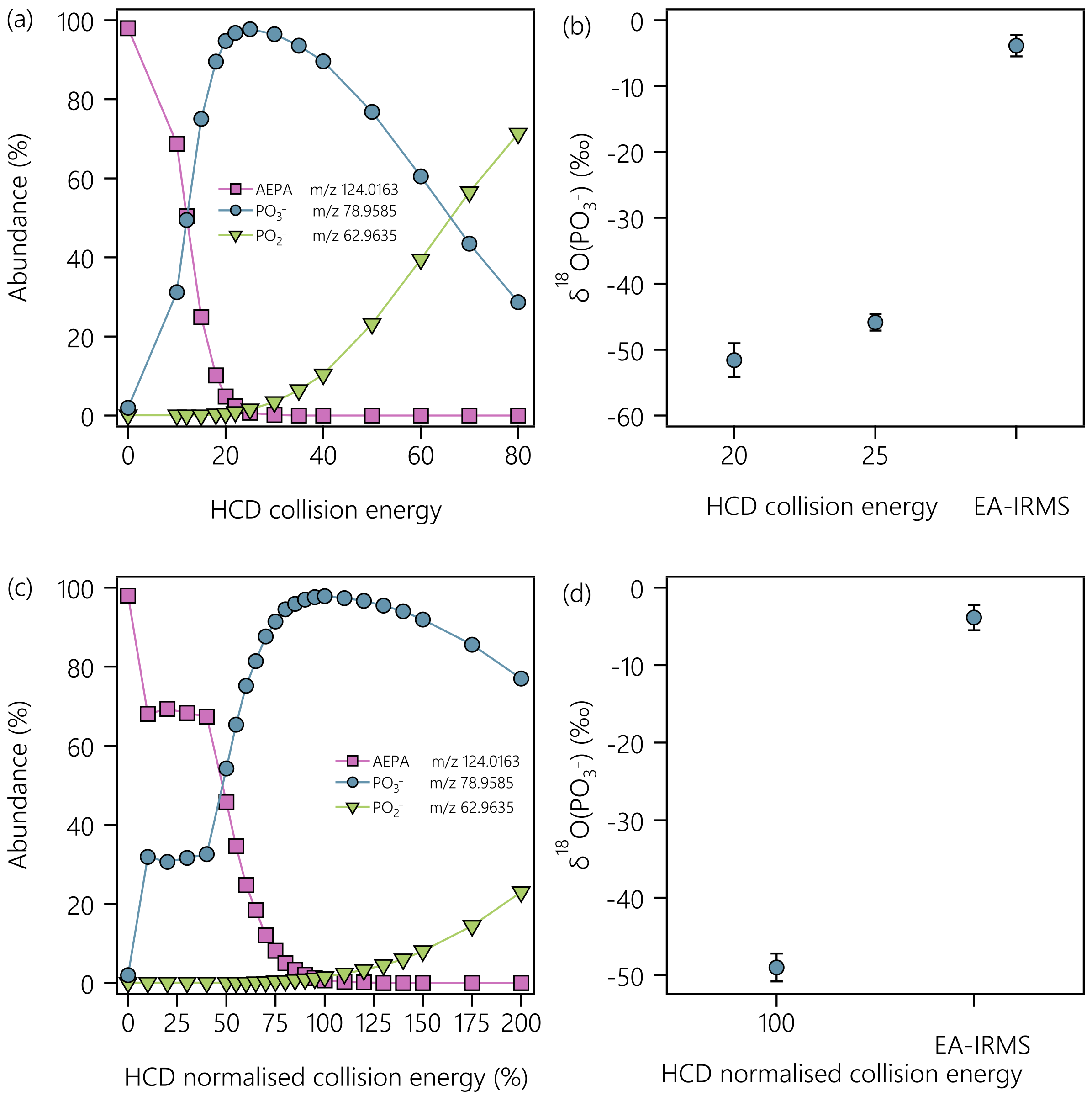

Figure S13 Relative abundances of aminoethylphosphonic acid (AEPA) ions and its two phosphonate fragments PO_3^- and PO_2^- at different (a) CE and (c) NCE energies used in the HCD cell. $\delta^{18}O(PO_3^-)$ values at different (b) CE and (d) NCE energies and the corresponding value from EA-IRMS measurements.

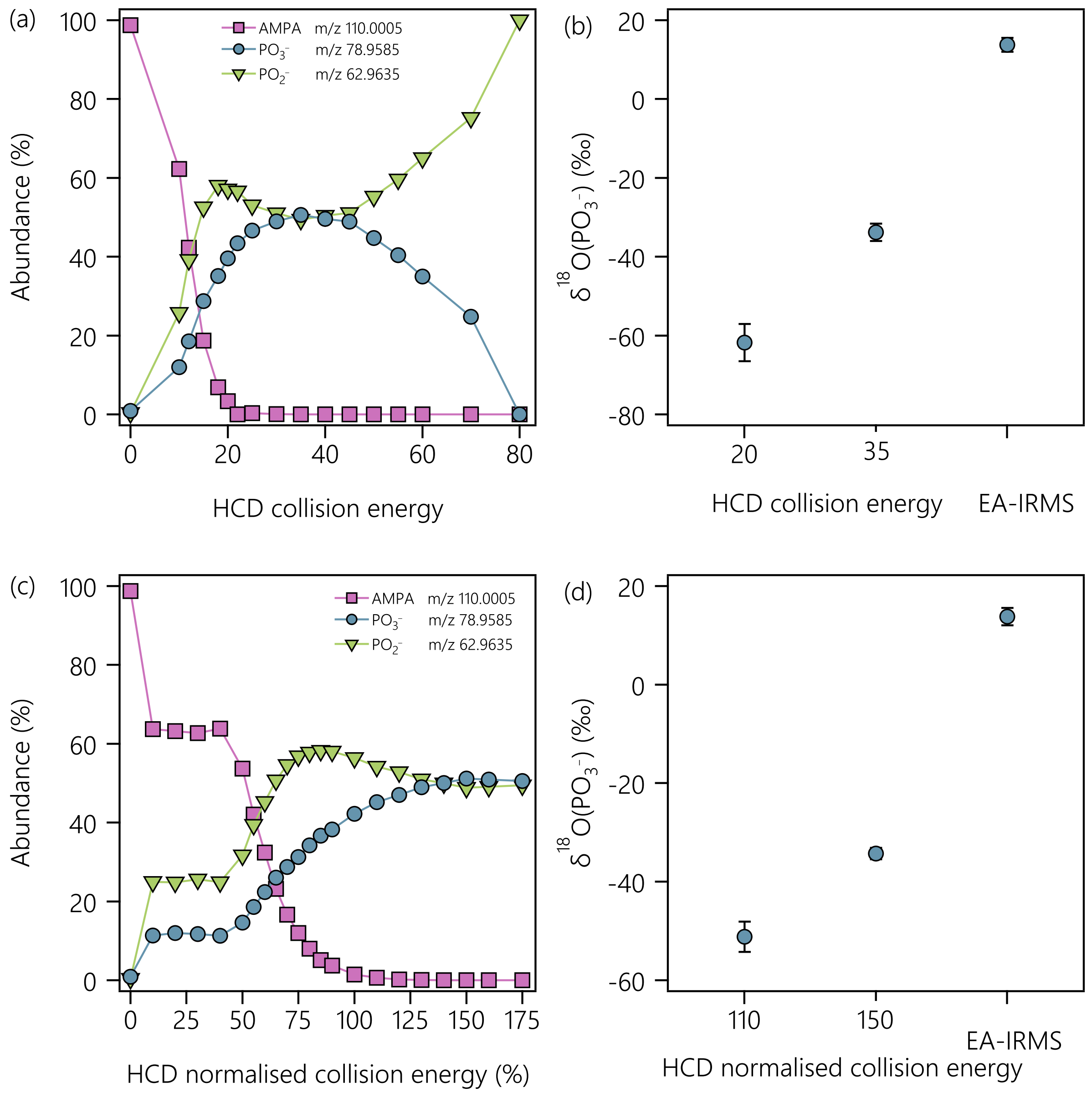

Figure S14 Relative abundances of aminomethylphosphonic acid (AMPA) ions and its two phosphonate fragments PO_3^- and PO_2^- at different (a) CE and (c) NCE energies used in the HCD cell. $\delta^{18}O(PO_3^-)$ values at different (b) CE and (d) NCE energies and the corresponding value from EA-IRMS measurements.

S2.4 Effects of Aqueous Matrix and Co-solutes

S2.4.1 Oxygen Exchange During Measurement

To asses the extent of oxygen atoms exchange between water and phosphate in the ionization source, we measured phosphate samples containing 5 vol-% water fraction with different $\delta^{18}O$ mol%, Figure S15. These tests showed that the exchange of oxygen atoms in the ionization source has no significant effect on the determined $\delta^{18}O(PO_4)$.

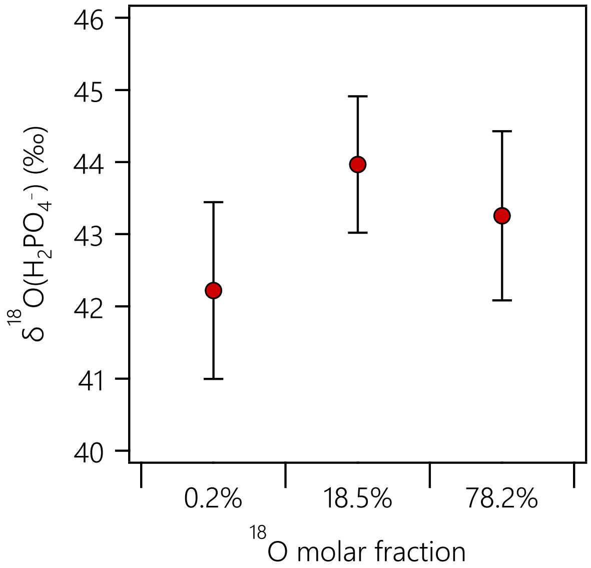

Figure S15 Variations of $\delta^{18}O$ values in phosphate standard G containing 5% (v/v) water with different ^{18}O molar fraction. No statistically significant variation in the values can be observed with increasing ^{18}O mol%.

S2.5 Sulfate

HSO_4^--ions fall within the chosen quadrupole mass range of 95–105 m/z. To ensure that sulfate (HSO_4^-) and phosphate ($H_2PO_4^-$) peaks of interested (namely ^{16}O and ^{18}O) are baseline separated and can thus be measured independently, we looked at the spectra. Figure S16 shows that at the chosen resolution of 70'000 the peaks are well separated.

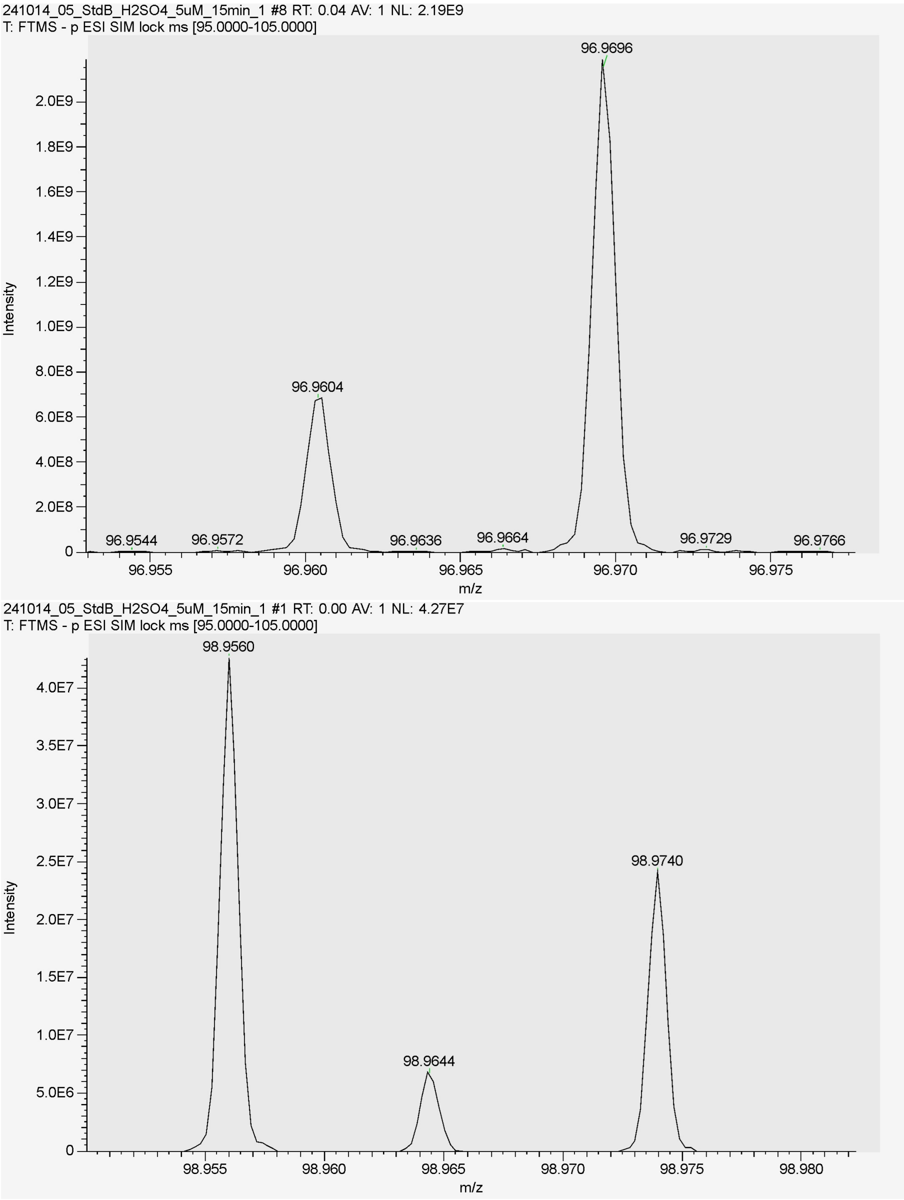

Figure S16 Spectrum of 50 μM $H_2PO_4^-$ with 5 μM HSO_4^- in the range of (a) ^{16}O and (b) ^{18}O at resolution 70'000. The following isotopologues are shown in order of appearance: $H^{32}S^{16}O_4^-$, $H_2P^{16}O_4^-$, $H^{33}S^{16}O_4^-$, $H^{32}S^{18}O^{16}O_3^-$, and $H_2P^{18}O^{16}O_3^-$.

S2.6 Sample purification procedure for selective phosphate extraction from aqueous solutions with metal-organic frameworks

S2.6.1 Sample Purification Procedure

The phosphate extraction and enrichment procedure by MOF for Orbitrap MS compatible samples consists of four steps: (1) phosphate sorption to MOF, (2) phosphate desorption from MOF, (3) cation exchange and (4) dilution in Orbitrap MS compatible solvent, here methanol.

First, the phosphate concentration in the sample is measured by UV-Vis as previously described (see Section S1.3.1). For the phosphate extraction, 0.75 mL of a 0.760 g/L MOF stock in 0.1 mM NaCl (solution II) were added to phosphate samples of varying concentrations (solution I). The samples are shaken overhead for 24 hours, before they are centrifuged for 15 min at 2655 rcf and 25°C (Eppendorf^{TM} 5427 R Model) to allocate MOF nanoparticles at the bottom of the sample container. 1.4 mL supernatant is then carefully removed leaving 100μL in the vial to ensure that no MOFs are lost. Phosphate concentration in the supernatant is measured and the amount of adsorbed phosphate calculated. To recover phosphate from MOFs, 1 mL Na_2CO_3 (solution IV) is applied to the 100 μL MOF slurry (solution III). The maximal carbonate concentration of 400 μmol/mL is tolerated to ensure the feasibility of the cation exchange. The sample are shaken overhead for 1–11 days. The samples are again centrifuged (15min, 2655 rcf and 25°C), the supernatant containing phosphate (solution VI) removed and the phosphate concentration measured. The supernatant V was subject to cation exchange with membranes. Cation exchange membranes of 1 cm^2 conditioned in nitric acid for minimum 24 h and washed with water prior to usage were applied. The samples were then subject to filtration with a 0.2 μm syringe filter before being diluted in methanol to reach a phosphate concentration of 50 μM.

Table S5 Properties of solutions and suspensions used in the sample purification procedure for nitrate and sulfate moval as described in Figure 3 of the main manuscript.

		Solution or suspension number						
Property	(units)	I	II	III	IV	V	VI	VII
Volume	(mL)	0.75	0.75	0.1	1	1	$\sim$ 0.9	varies
PO_4^{3-}	(μM)	660	-	-	n.d.[b]	250	180	50
	(μmol)	0.66	-	-	n.d.	0.24	0.2	-[d]
Zr-MOF	(g/L)	-	0.76	5.6	-	-	-	-
Na^{+}[a]	(mM)	-	0.1	0.05	160–680	160–680	n.d.[e]	-
	(mmol)	-	0.0001	$\ll$0.0001	0.16–0.68	0.16–0.68	n.d.[e]	-
CO_3^{2-}	(mM)	0	0	0	80–340	80–340	n.d.[f]	n.d.[f]

[a] from NaCl or Na_2CO_3 [b] n.d. = not determined
[c] n.a. = not applicable, depends on volume
[d] depends on volume [e] n.d. = not determined, as cation exchange membranes with average Na^+ removal per membrane of 41.41 ± 11.81 were applied so amount would reach 0 μmol
[f] not determined, but concentration will not be comparable to solution V

S2.6.2 Competing Anions

Table S6 Amounts and fractions of nitrate, sulfate, and phosphate, before (solution I, Figure 3 in main manuscript), during (supernatant of III), and after (solution VI) MOF-based sample purification procedure. Percentages indicate the fraction of the remaining and anion mass for supernatant of V and solution VI. $\delta^{18}O(PO_4)$ values were determined of (1) the NaH_2PO_4 salt used to prepare solution I and (2) solution VI.

sampling point	Nitrate		Sulfate		Phosphate		$\delta^{18}O(PO_4)$
	μmol	%	μmol	%	μmol	%	‰
Solution I	0.151	100[a]	0.847	100	0.656	100	23.9 ± 2.8
Supernatant of III	0.147	98	0.805	95	0.312	48	n.d.[b]
Solution VI	0.008	5	0.066	8	0.200	31	25.3 ± 3.0

[a] initial conditions [b] n.d. = not determined

S2.6.3 Extraction

Concentrated stock suspensions of Zr-BDC were prepared in 0.1 mM NaCl in a nanotechnology enclosure (Labconco, X Pert Nano). The MOFs particles were dispersed in solution through sonication for 1 minute. The concentrated stock suspensions were then diluted in 0.1 mM NaCl, resulting in suspensions with a concentration of 14.7 mg/L and 760 mg/L Zr-BDC. To determine the phosphate adsorption capacity of Zr-BDC in mmol/g, we combined 750 μL of diluted MOF suspension with 750 μL of a NaH_2PO_4 aqueous solution with concentrations ranging from 1 μM to 70 μM and 0.4 mM to 8 mM for low and high conentration of MOF respectively. The samples were then placed overnight on a rotary mixer and centrifuged (15 min, 25°C, 2655 rcf). After centrifuging, the phosphate concentration was measured in the supernatant and the different phosphate starting stock solutions using the Malachite green method described above. The adsorbed and aqueous phosphate concentration was then calculated.

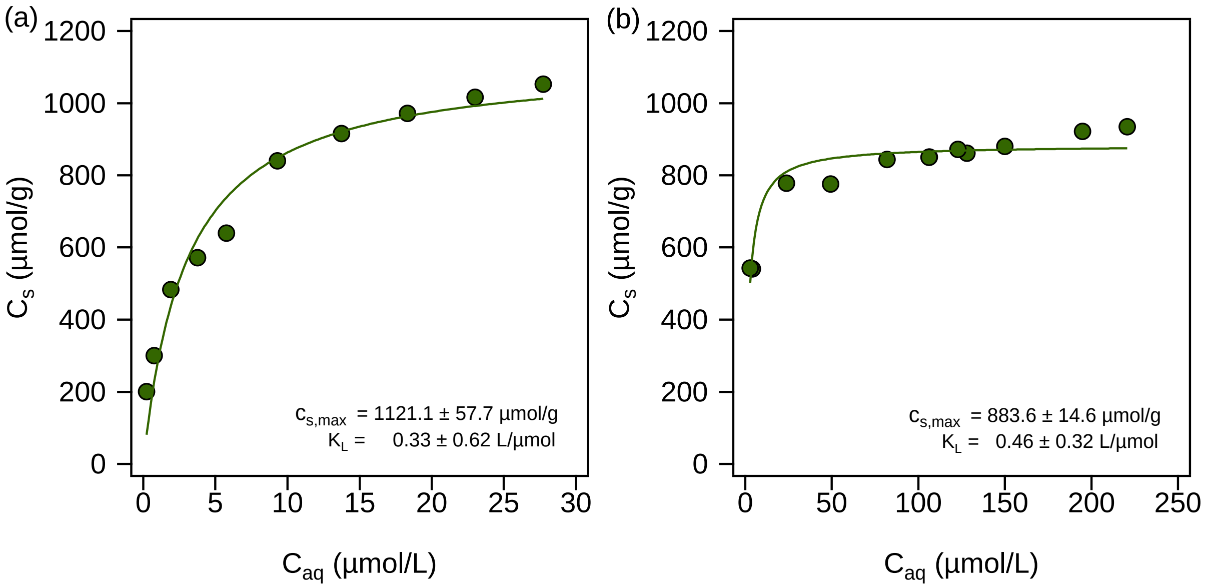

Figure S17 Langmuir sorption isotherms of solutions containing (a) 7.4 mg/L and (b) 380 mg/L Zr-BDC MOFs with varying phosphate concentration. A fit to the Langmuir model ($c_s = (c_{s,max} \cdot K_L \cdot c_{aq})/(1 + K_L \cdot c_{aq})$) was used to determine the maximum adsorbed amount of phosphate ($c_{s,max}$ in μmol/g) and affinity constant K_L (see data in figure panels). $c_{s,max}$-values for high and low MOF loadings of 1.1 mmol/g (107 mg/g) and 0.9 mmol/g (84 mg/g), respectively, correspond well to earlier data from Lin et al.[8] (85 mg/g), whereas the K_L of our study indicate a higher sorption affinity (3 L/mg in this study vs. approx. 0.1 L/mg).

S2.6.4 Recovery

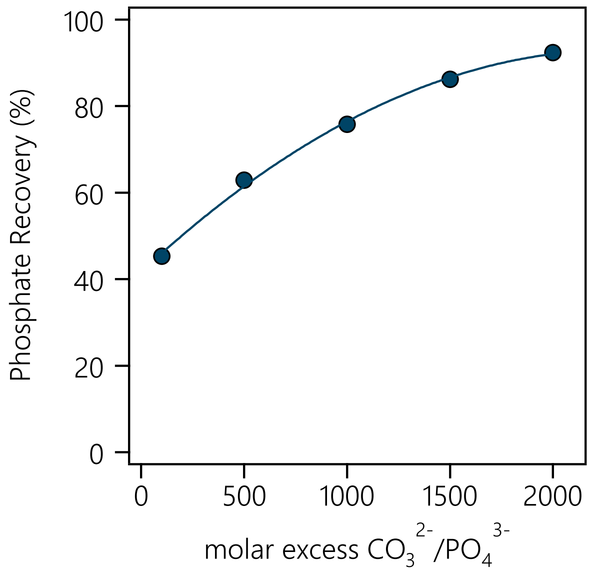

Figure S18 Phosphate recovery at different molar excess of carbonate applied to MOF suspension III (see Figure 3 of the main manuscript).

S2.6.5 Cation Removal

Table S7 Na^+ concentration before and after exposure of solutions to cation exchange membranes and amount of Na^+ removed per mL after each cation-exchange step. One fresh membrane was used per 1 mL sample solution. The average Na^+ removal per membrane was 41.41 ± 11.81 μmol.

Number of step	Concentration in sample (mM)	Na^+ removal per mL (μmol)
initial	869.77	-
1	829.26	39.51
2	779.02	50.24
3	742.69	36.33
4	721.45	21.24
5	663.64	57.80
6	616.16	47.48
7	578.94	37.23

References

[1] Shearer, G. C.; Chavan, S.; Bordiga, S.; Svelle, S.; Olsbye, U.; Lillerud, K. P. Defect engineering: tuning the porosity and composition of the metal–organic framework UiO-66 via modulated synthesis. *Chemistry of Materials* **2016**, *28*, 3749–3761.

[2] Ohno, T.; Zibilske, L. Determination of low concentrations of phosphorus in soil extracts using malachite green. *Soil Science Society of America Journal - SSSAJ* **1991**, *55*, https://doi.org/10.2136/sssaj1991.

[3] DIN EN ISO 14911; Wasserbeschaffenheit - Bestimmung der gelösten Kationen Li^{+}, Na^{+}, NH_4^{+}, K^{+}, Mn^{2+}, Ca^{2+}, Mg^{2+}, Sr^{2+} und Ba^{2+} mittels Ionenchromatographie - Verfahren für Wasser und Abwasser. https://www.dinmedia.de/en/standard/din-en-iso-14911/23050526, 1999.

[4] DIN EN ISO 10304-1; Wasserbeschaffenheit - Bestimmung von gelösten Anionen mittels Flüssigkeits-Ionenchromatographie - Teil 1: Bestimmung von Bromid, Chlorid, Fluorid, Nitrat, Nitrit, Phosphat und Sulfat. https://www.dinmedia.de/de/norm/din-en-iso-10304-1/117316025, 2009.

[5] Gröning, M. Some pitfalls in the uncertainty evaluation of isotope delta reference materials. *Accred. Qual. Assur.* **2023**, *28*, 101–114, https://doi.org/10.1007/s00769-022-01527-6.

[6] Hilkert, A.; Böhlke, J. K.; Mroczkowski, S. J.; Fort, K. L.; Aizikov, K.; Wang, X. T.; Kopf, S. H.; Neubauer, C. Exploring the potential of electrospray-Orbitrap for stable isotope analysis using nitrate as a model. *Anal. Chem.* **2021**, *93*, 9139, https://doi.org/10.1021/acs.analchem.1c00944.

[7] Bernet, N. M.; Hofstetter, T. B. Advances in oxygen isotope analysis of phosphate by electrospray orbitrap mass spectrometry for studying the microbial metabolism of microorganisms. *CHIMIA* **2024**, *78*, 256–260, https://doi.org/10.2533/chimia.2024.256.

[8] Lin, K.-Y. A.; Chen, S.-Y.; Jochems, A. P. Zirconium-based metal organic frameworks: Highly selective adsorbents for removal of phosphate from water and urine. *Mater. Chem. Phys.* **2015**, *160*, 168–176, https://doi.org/10.1016/j.matchemphys.2015.04.021.
